# Selective Whole-Genome Amplification Is a Robust Method That Enables Scalable Whole-Genome Sequencing of* Plasmodium vivax* from Unprocessed Clinical Samples

**DOI:** 10.1128/mBio.02257-16

**Published:** 2017-02-07

**Authors:** Annie N. Cowell, Dorothy E. Loy, Sesh A. Sundararaman, Hugo Valdivia, Kathleen Fisch, Andres G. Lescano, G. Christian Baldeviano, Salomon Durand, Vince Gerbasi, Colin J. Sutherland, Debbie Nolder, Joseph M. Vinetz, Beatrice H. Hahn, Elizabeth A. Winzeler

**Affiliations:** a Division of Infectious Diseases, Department of Medicine, University of California, San Diego, La Jolla, California, USA; b Departments of Medicine and Microbiology, Perelman School of Medicine, University of Pennsylvania, Philadelphia, Pennsylvania, USA; c Universidade Federal de Minas Gerais, Pampulha, Belo Horizonte, MG, Brazil; d Center for Computational Biology and Bioinformatics, UC San Diego, La Jolla, California, USA; e Universidad Peruana Cayetano Heredia, Lima, Peru; f U.S. Naval Medical Research Unit No. 6, Bellavista, Callao, Peru; g Public Health England Malaria Reference Laboratory, Faculty of Infectious and Tropical Diseases, London School of Hygiene and Tropical Medicine, London, United Kingdom; h Division of Host-Microbe Systems & Therapeutics, Department of Pediatrics, UC San Diego, La Jolla, California, USA; NIAID/NIH

## Abstract

Whole-genome sequencing (WGS) of microbial pathogens from clinical samples is a highly sensitive tool used to gain a deeper understanding of the biology, epidemiology, and drug resistance mechanisms of many infections. However, WGS of organisms which exhibit low densities in their hosts is challenging due to high levels of host genomic DNA (gDNA), which leads to very low coverage of the microbial genome. WGS of *Plasmodium vivax*, the most widely distributed form of malaria, is especially difficult because of low parasite densities and the lack of an *ex vivo* culture system. Current techniques used to enrich *P. vivax* DNA from clinical samples require significant resources or are not consistently effective. Here, we demonstrate that selective whole-genome amplification (SWGA) can enrich *P. vivax* gDNA from unprocessed human blood samples and dried blood spots for high-quality WGS, allowing genetic characterization of isolates that would otherwise have been prohibitively expensive or impossible to sequence. We achieved an average genome coverage of 24×, with up to 95% of the *P. vivax* core genome covered by ≥5 reads. The single-nucleotide polymorphism (SNP) characteristics and drug resistance mutations seen were consistent with those of other *P. vivax* sequences from a similar region in Peru, demonstrating that SWGA produces high-quality sequences for downstream analysis. SWGA is a robust tool that will enable efficient, cost-effective WGS of *P. vivax* isolates from clinical samples that can be applied to other neglected microbial pathogens.

## IMPORTANCE

Malaria is a disease caused by *Plasmodium* parasites that caused 214 million symptomatic cases and 438,000 deaths in 2015. *Plasmodium vivax* is the most widely distributed species, causing the majority of malaria infections outside sub-Saharan Africa. Whole-genome sequencing (WGS) of *Plasmodium* parasites from clinical samples has revealed important insights into the epidemiology and mechanisms of drug resistance of malaria. However, WGS of *P. vivax* is challenging due to low parasite levels in humans and the lack of a routine system to culture the parasites. Selective whole-genome amplification (SWGA) preferentially amplifies the genomes of pathogens from mixtures of target and host gDNA. Here, we demonstrate that SWGA is a simple, robust method that can be used to enrich *P. vivax* genomic DNA (gDNA) from unprocessed human blood samples and dried blood spots for cost-effective, high-quality WGS.

## INTRODUCTION

Malaria is a mosquito-borne infection caused by protozoan parasites of the *Plasmodium* genus. Of the six *Plasmodium* species known to infect humans (1–3), *P. vivax* is the most widely distributed, causing approximately half of all clinical cases of malaria outside Africa (4). Whole-genome sequencing (WGS) of *Plasmodium* parasites from clinical samples has revealed important insights into the biology, epidemiology, and mechanisms of drug resistance of malaria (5–12). For *P. vivax*, WGS of clinical isolates has the potential to uncover mechanisms underlying some of the unique aspects of this parasite’s biology, such as distinguishing between reinfection and relapse due to activation of dormant liver parasites. However, WGS of *P. vivax* from clinical samples is challenging, mainly due to low parasite densities in clinical samples compared to those of *Plasmodium falciparum* and the lack of a robust *ex vivo* culture system.

Multiple techniques have been developed to enrich *Plasmodium* genomic DNA (gDNA) from clinical samples, including leukocyte depletion (13–15), hybrid selection with RNA baits (16, 17), short-term *ex vivo* culture (18), adaptation to growth in splenectomized monkeys (9, 19), and single-cell sequencing (20). The majority of these techniques require significant labor and resources. While leukocyte depletion is the most cost-effective, it requires sample processing within 6 h of sample collection, which is not feasible at many field sites and is not always effective. Two recent studies that performed WGS on over 400 clinical isolates of *P. vivax* (15, 17) employed hybrid selection and leukocyte depletion to enrich *P. vivax* gDNA from clinical samples. Pearson et al. used leukocyte depletion on 292 clinical samples and had to eliminate 144 (49%) of their samples from further population genetics analysis due to low quality, which often occurs due to contaminating human DNA (15). Hupalo et al. used hybrid selection to enrich their samples, with 31 out of 170 sequences (21%) removed from further analysis due to low quality (17). Although more frequently successful, the hybrid selection technique requires either expensive synthetic RNA baits or a large amount of pure *P. vivax* DNA to create the RNA baits, which is difficult to obtain. In addition, hybrid selection can introduce bias, since it is approximately half as efficient at capturing regions with GC contents of >50% (16).

**FIG 1:**
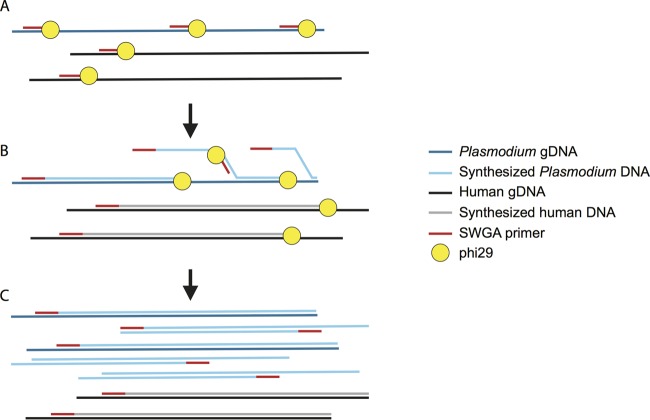
Selective whole-genome amplification (SWGA) of *Plasmodium vivax* genomic DNA (gDNA) from human blood samples. (A) SWGA primers bind frequently to *Plasmodium vivax* gDNA and infrequently to human gDNA. (B) When phi29 encounters double-stranded gDNA, it displaces the newly synthesized strand, opening new primer binding sites on the synthesized gDNA, leading to selective amplification of templates with frequent primer binding sites. (C) Post-SWGA, the percentage of *P. vivax* DNA has increased relative to the percentage of host DNA.

An alternative method is selective whole-genome amplification (SWGA). SWGA has been used to enrich submicroscopic DNA levels of the ape *Plasmodium* parasites, *P. reichenowi* and *P. gaboni*, from whole-blood samples (21, 22) and *P. falciparum* genomes from dried blood spots (23) for WGS. SWGA preferentially amplifies the genomes of pathogens from complex mixtures of target and host DNA (Fig. 1) (24). SWGA does not require separation of target DNA from background DNA, making it an attractive option for pathogens that cannot be amplified in culture. DNA amplification is carried out by the highly processive, strand-displacing phi29 DNA polymerase and a set of pathogen-specific primers that target short (6 to 12 nucleotide) motifs that are common in the pathogen genome and uncommon in the host genome. The strand displacement function of phi29 results in the amplification of genomic regions where primers bind frequently, leading to the preferential amplification of genomes with frequent primer-binding sites. Here, we show that SWGA efficiently enriches *P. vivax* gDNA from unprocessed human blood samples and dried blood spots for cost-effective, high-quality whole-genome sequencing.

## RESULTS

### Primer design and optimization using *P. vivax*-infected whole-blood samples.

To perform SWGA on *P. vivax* gDNA from unprocessed human blood samples, we designed primers that specifically amplified this parasite’s DNA using a previously published approach for *P. falciparum* (22). Briefly, we identified the most frequently occurring motifs of 6 to 12 nucleotides in length in the *P. vivax* Salvador-1 (Sal-1) reference genome. We selected the top 10,000 primers of each length, yielding a total of 70,000 primers for further analysis. We filtered these primers based on characteristics such as melting temperature (18 to 32°C), ability to homodimerize (no greater than 3 consecutive matches), binding frequencies on the human genome and Sal-1 genome (less frequent than once every 500,000 bp and more frequent than once every 50,000 bp, respectively), and infrequent binding of the human mitochondrial genome (less than 4 binding sites). Next, we removed primers predicted to bind the Sal-1 subtelomeres. These filters resulted in a pool of 222 primers. We separated these primers into 6 sets that were predicted not to form heterodimers and identified the top set (pvset1) of 10 primers using a selection algorithm described previously (22). gDNA from an unprocessed *P. vivax*-infected whole-blood sample (MRL2) was subjected to SWGA with primer set pvset1 prior to shotgun sequencing (see Fig. S1 in the supplemental material). SWGA significantly increased the percentage of reads that mapped to the *P. vivax* Sal-1 reference genome, from 0.7% to 73.5% (see Fig. S1A), and improved the genome coverage obtained from ~80 million bp of sequencing from 1.5% to 58% (see Fig. S1B).

10.1128/mBio.02257-16.1 FIG S1  Testing of SWGA primer sets on an unprocessed, *P. vivax*-infected blood sample. (A) Unamplified DNA (black) and DNA amplified with SWGA primer set pvset1 (blue) was sequenced on a MiSeq (Illumina). The percentage of MiSeq reads that mapped to the *P. vivax* Sal-1 reference genome in Geneious (version 9.1) was plotted for both the unamplified and SWGA-amplified sample. (B) Rarefaction analysis compares the ≥1× *P. vivax* genome coverage relative to total sequencing depth (in millions of base pairs sequenced) with and without SWGA. Download FIG S1, TIF file, 69.2 MB. Copyright © 2017 Cowell et al. 2017 Cowell et al. This content is distributed under the terms of the Creative Commons Attribution 4.0 International license.

**FIG 2:**
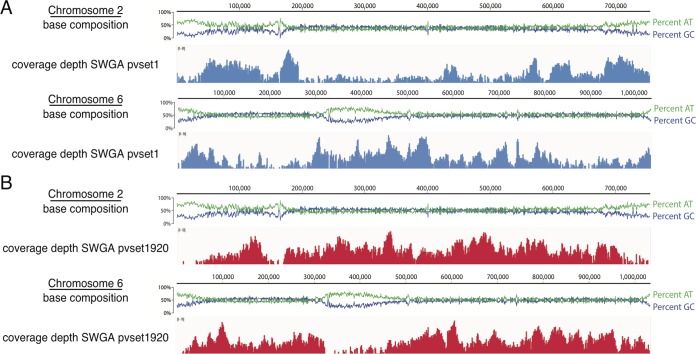
*Plasmodium vivax* chromosomal coverage following SWGA using primer set pvset1 (A) or pvset1920 (B). The base compositions of chromosomes 2 and 6 were visualized in Geneious (version 9.1) using the *P. vivax* Sal-1 reference genome; green and blue lines represent percentages of AT and GC content, respectively, plotted for 25-bp windows across the chromosome (scale shown above the graph). Shown in blue and red below are the corresponding MiSeq read coverage depths using primer sets pvset1 and pvset1920, respectively. Coverage plots were generated using IGVTools (version 2.3.40) and are shown on a log scale with maximum read depth indicated in the upper left corner of the plot.

We observed that the genome coverage obtained per base pair sequenced was lower than that achieved with SWGA of *P. falciparum* gDNA using the same primer set design methods (22). Visual inspection of the *P. vivax* genome coverage from samples subjected to SWGA revealed that coverage gaps were typically in regions with comparatively higher GC content (Fig. 2A). The *P. falciparum* genome is extremely AT rich, with only 19.4% of bases consisting of G’s or C’s, while the GC content for the *P. vivax* genome is 42.3% (9). The *P. vivax* genome also has an isochore structure: internal chromosomal areas have a high GC content and subtelomeres and centromeres have lower GC contents (25). Since phi29 DNA polymerase pauses more frequently during strand displacement and primer extension in regions with a high GC content of DNA (26), we hypothesized that differences in base composition could explain the more uneven amplification of the *P. vivax* genome compared to that of *P. falciparum*.

**FIG 3:**
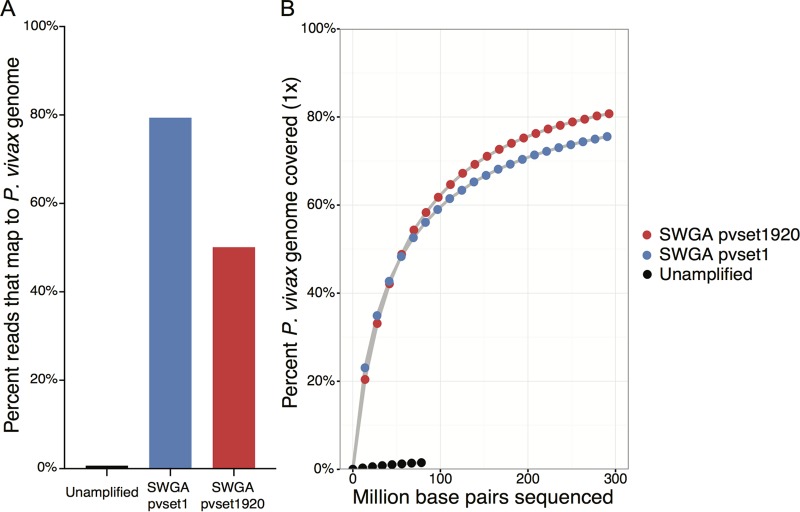
Testing of SWGA primer sets on DNA from an unprocessed, *P. vivax*-infected blood sample. (A) Unamplified DNA (black) and DNA amplified with SWGA primer set pvset1 (blue) or pvset1920 (red) was sequenced on a MiSeq (Illumina). The percentages of MiSeq reads that mapped to the *P. vivax* Sal-1 reference genome in Geneious (version 9.1) (39) were plotted for both unamplified and SWGA-amplified samples. (B) The 1× *P. vivax* genome coverage is shown relative to the total sequencing depth (in millions of base pairs sequenced) for samples subjected to SWGA with pvset1920 or pvset1 and for unamplified DNA.

We thus designed primer sets specifically targeting regions of the *P. vivax* Sal-1 reference genome with high GC content and poor coverage using the *swga* program (https://github.com/eclarke/swga; unpublished data), a program that identifies and scores SWGA primer sets (see Fig. S2 in the supplemental material). Primers were designed to bind regions of the *P. vivax* Sal-1 genome that had even AT/GC composition, were longer than 195,000 bp, and had low sequence coverage when amplified with pvset1. We identified 1,939 primer sets (consisting of up to 15 primers) with minimal human genome binding and maximal *P. vivax* genome binding and scored them based on evenness of binding, as well as mean distance between primer binding sites in the foreground and background genomes. The primer set with the best score, pvset1920, was chosen for subsequent testing. SWGA of an unprocessed human blood sample with pvset1920 yielded overall superior *P. vivax* genome coverage compared to that of SWGA with pvset1 (Fig. 3). Visual inspection of these post-SWGA *P. vivax* sequences revealed that pvset1920 achieved improved coverage particularly in regions with high GC content (Fig. 2B), with troughs in coverage in genomic regions of lower GC content, which include the centromeres and subtelomeres.

10.1128/mBio.02257-16.2 FIG S2  Flowchart describing the process of SWGA primer set selection for *Plasmodium vivax*. *, selected regions are >195,000 bp in length with an average 48.5 to 50.6% GC composition. The *swga* program is available on (https://github.com/eclarke/swga; unpublished data). Download FIG S2, TIF file, 31.4 MB. Copyright © 2017 Cowell et al. 2017 Cowell et al. This content is distributed under the terms of the Creative Commons Attribution 4.0 International license.

Having developed a method that worked well for SWGA of *P. vivax* gDNA from whole blood, we tested whether the method could also be applied to gDNA extracted from dried blood spot samples. Dried blood spots are a common method of storing patient and parasite DNA that utilizes a smaller volume of blood and does not require immediate cold storage. DNA extracted from dried blood spots can have variable quality depending on the method of collection and storage (27). SWGA has been used to enrich *P. falciparum* DNA from dried blood spots for WGS (23), with an average of 48.1% ± 3.5% of the genome covered at ≥5× for samples with an average parasite density of 73,601 parasites/µl ± 19,399 (1.5% parasitemia). Since *P. vivax* clinical samples generally have lower parasite densities, we wondered if it would be feasible to obtain significant genome coverage on *P. vivax* from dried blood spots with SWGA. We extracted DNA from blood spots obtained from symptomatic patients in Peru and performed SWGA with pvset1920, achieving 73% and 42% genome coverage at 1× on initial testing for samples with a high (sample C, 56,790 parasites/µl; 1.1%) and low (sample L, 2,572 parasites/µl; 0.05%) level of parasitemia, respectively (see Fig. S3 in the supplemental material).

10.1128/mBio.02257-16.3 FIG S3  Testing of SWGA primer set pvset1920 on DNA from *P. vivax* patient-derived dried blood spots. Genomic DNA from two dried blood spots with either a relatively high (sample C, 56,790 parasites/µl) or low (sample L, 2,572 parasites/µl) parasite density was subjected to SWGA with pvset1920 and sequenced on a MiSeq (Illumina). The percentages of the *P. vivax* genome with ≥1× coverage are shown relative to total sequencing depth (in millions of base pairs sequenced). Download FIG S3, TIF file, 10.6 MB. Copyright © 2017 Cowell et al. 2017 Cowell et al. This content is distributed under the terms of the Creative Commons Attribution 4.0 International license.

We finally tested whether an enzymatic digest to remove contaminating human DNA could further improve *P. vivax* genome coverage. Modification-dependent restriction endonucleases (MDREs), such as MspJI and FspEI, which specifically recognize cytosine C-5 methylation or hydroxymethylation (28), have been used to selectively degrade human DNA in *P. falciparum* clinical samples. Enzyme digest of DNA extracted from clinical samples with >80% human contamination has previously been shown to enrich *P. falciparum* DNA ~ninefold for more efficient WGS (29). However, when we performed a digest with MspJI and FspEI enzymes on gDNA extracted from whole blood obtained from patients with *P. vivax* infection, we observed either no change or markedly decreased genome coverage in the 5 enzyme-digested samples (see Fig. S4 in the supplemental material).

10.1128/mBio.02257-16.4 FIG S4  Comparison of SWGA following either a mock digestion or methylation-dependent restriction digest. Genomic DNA extracted from five *P. vivax*-infected patient samples was subjected to a mock digest or digest with the methylation-dependent restriction enzymes MspJI and FspEI. The percentage of the *P. vivax* reference genome covered (1×) by mapping 200,100 MiSeq (Illumina) reads (approximately 25 million bp of sequencing depth) from each sample with or without digest is shown. Statistical significance was calculated using a two-tailed *t* test. Download FIG S4, TIF file, 31.4 MB. Copyright © 2017 Cowell et al. 2017 Cowell et al. This content is distributed under the terms of the Creative Commons Attribution 4.0 International license.

### SWGA and WGS of *P. vivax* from patient samples.

To test the utility of SWGA for variant calling and population genetics analysis of *P. vivax* from unprocessed clinical blood samples, we used primer set pvset1920 to perform SWGA on *P. vivax* gDNA from 18 whole-blood and 4 dried blood spot samples collected from symptomatic patients with *P. vivax* infection in Peru. Since the whole-blood samples had not been leukocyte filtered, they had significant contamination with human DNA, with less than 1.5% of reads mapping to the Sal-1 reference genome in unamplified samples (not shown). For all samples, SWGA significantly increased the proportion of reads that mapped to the *P. vivax* Sal-1 reference genome, resulting in higher genome coverage and higher percentages of callable total- and core-genome regions (covered by ≥5 reads) (Table 1). Comparison of the SWGA-amplified samples to 10 leukocyte-filtered samples from a field study in Peru which were sequenced to a similar depth (1.5 ± 0.2 billion bp sequenced for SWGA samples versus 1.5 ± 0.5 billion bp for leukocyte-filtered samples) showed that SWGA yields a twofold increase in the percentage of sequencing reads that map to the* P. vivax* genome and an average 5× *P. vivax* core-genome coverage of 60.1% ± 26.0%, compared to 43.7% ± 41.4% for leukocyte-filtered samples (10). For the 4 dried blood spot samples, we achieved an average 5× core-genome coverage of 54.0% ± 34.6%.

**TABLE 1:** Sequencing statistics for *P. vivax* sequences from clinical samples that underwent selective whole-genome amplification^a^

Enrichment technique(sample)	No. of parasites/µl(% parasitemia)^b^	Total no. of bp sequenced (billions)	Reads aligned to *P. vivax*reference (%)	Mean coverage (×)	% callable^c^:	
					Genome	Core genome
SWGA (18 whole-blood samples)						
1	45,680 (0.9)	1.42	89.5	37.1	71.7	83.6
2	34,268 (0.7)	1.68	80.9	37.9	72.9	85.1
3	28,474 (0.6)	1.41	88.3	30.4	66.6	76.3
4	25,680 (0.5)	1.50	65.1	13.8	40.2	42.1
5	19,241 (0.4)	2.00	71.9	44.1	79.9	95.4
6	13,064 (0.3)	1.84	83.7	43.8	76.1	90.4
7	11,438 (0.2)	1.52	79.5	20.3	32.7	34.5
8	9,961 (0.2)	1.36	75.4	31.0	74.6	87.3
9	8,842 (0.2)	1.84	74.2	29.6	72.9	84.7
10	7,382 (0.1)	1.23	72.6	23.7	65.6	73.6
11	6,258 (0.1)	1.41	82.8	25.1	59.5	67.7
12	5,135 (0.1)	1.82	54.9	18.0	34.8	37.0
13	2,942 (0.06)	1.16	40.7	12.9	52.1	57.9
14	1,873 (0.04)	1.44	17.0	5.7	13.7	14.1
15	1,652 (0.03)	1.52	53.2	23.5	52.0	57.3
16	1,471 (0.03)	1.26	44.0	12.9	21.5	22.9
17	537 (0.01)	1.92	22.5	11.1	38.1	40.7
18	495 (0.01)	1.44	28.6	10.0	30.0	30.5
Avg ± SD	12,466 ± 13,107.2	1.5 ± 0.2	62.7 ± 23.2	23.9 ± 11.8	53.1 ± 20.9	60.1 ± 26.0
SWGA (4 dried bloodspot samples)						
DBS-4	50,330 (1.0)	1.97	79.4	34.8	74.4	87.4
DBS-3	35,730 (0.7)	1.91	46.8	20.9	69.5	80.3
DBS-2	5,932 (0.1)	0.93	11.0	3.0	25.8	24.2
DBS-1	3,885 (0.08)	1.57	17.7	4.2	23.2	24.1
Avg ± SD	23,962 ± 22,826.4	1.6 ± 0.5	38.7 ± 27.1	15.7 ± 13.1	48.2 ± 27.5	54.0 ± 34.6
Leukocyte filtration(10 whole-blood samples)						
Mdio01	NA	2.43	25.7	6.35	8.2	5.5
Mdio02	NA	0.80	16.2	1.65	1.3	0.6
Mdio03	NA	2.04	19.9	8.23	57.2	61.2
Mdio04	NA	1.52	14.2	1.97	18.7	9.6
Mdio05	NA	1.56	49	22.3	78.1	91.7
Mdio06	NA	1.62	55.4	28.1	79.6	93.4
Mdio07	NA	1.46	20.7	4.23	15.8	11.3
Mdio08	NA	1.50	30.3	10.6	67.3	74.9
Mdio09	NA	0.74	18.3	1.74	1.6	0.8
Mdio10	NA	1.26	43.5	16.1	76	88.1
Avg ± SD	NA	1.5 ± 0.5	31.4 ± 14.8	10.1 ± 9.2	40.4 ± 34.0	43.7 ± 41.4

a Sequencing statistics were determined using the Genome Analysis Toolkit’s (GATK) DepthofCoverage tool. The core genome was defined by coordinates determined in the large-scale *P. vivax* sequencing study by Pearson et al. (15). The leukocyte-filtered sequencing statistics presented here are from *P. vivax* clinical samples obtained from a previously published study in Peru (10).

b NA, not applicable.

c Covered by ≥5 reads.

**FIG 4:**
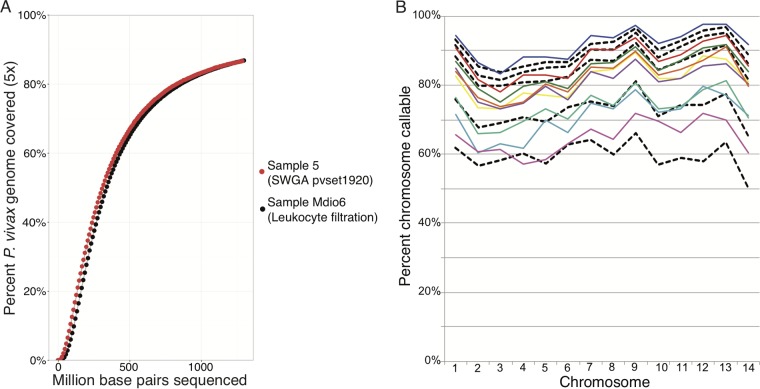
Comparison of *P. vivax* genome coverage generated from samples treated with SWGA and with leukocyte filtration. (A) *P. vivax* genome coverage (5×) is shown relative to total sequencing depth (in millions of base pairs sequenced) for a sample amplified with pvset1920 (sample 5) and for a leukocyte-filtered sample (Mdio6). Both samples were sequenced on an Illumina HiSeq. (B) The percentages of the 14 chromosomes that were callable (covered by ≥5 reads) for samples that underwent SWGA (colored lines) or leukocyte filtration (black dashed lines) were compared between multiple samples.

**FIG 5:**
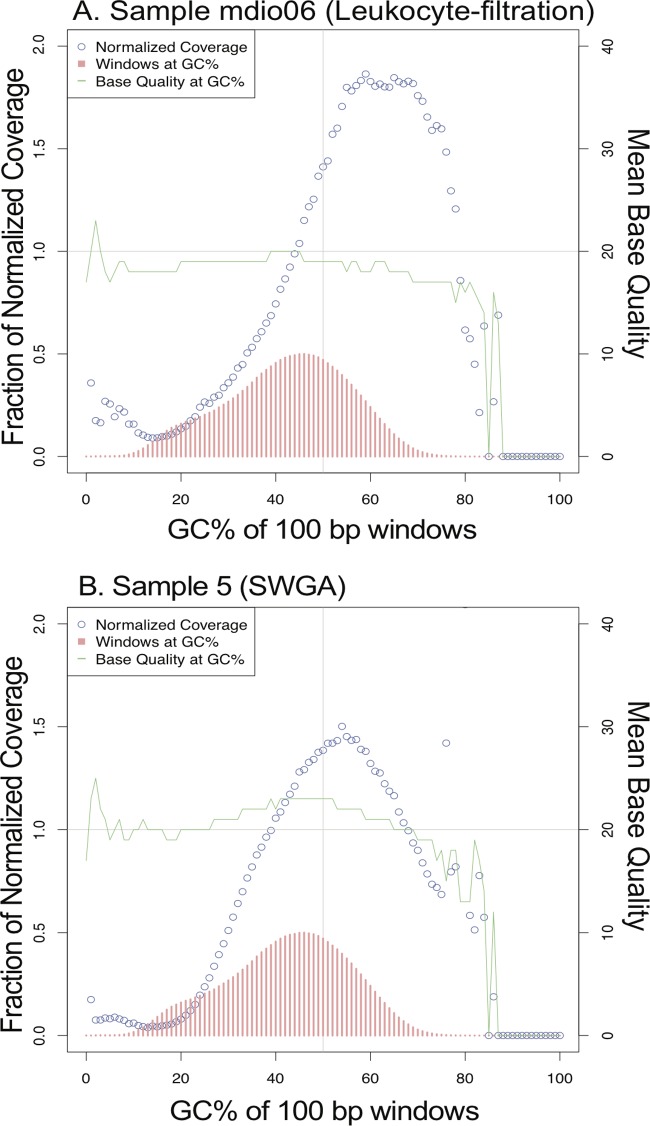
GC bias plots for *P. vivax* genomes generated following leukocyte filtration (A) or SWGA (B). GC bias plots were generated using Picard (version 2.0.1) on aligned sequencing reads from two different clinical samples that underwent leukocyte filtration (A) or selective whole-genome amplification (B). The number of windows with a given GC content are plotted with red bars; the base quality (green line) and normalized coverage (open blue circles) are plotted for different levels of GC content for both methods of *P. vivax* gDNA enrichment.

There was a trend toward improved mean coverage and percentage of the genome callable in samples with higher parasite densities (see Fig. S5 in the supplemental material). This is consistent with previous SWGA results for *P. falciparum* (22) and results from other *P. vivax*-enriching methods, such as hybrid selection (16). Samples subjected to SWGA yielded similar ≥5× genome coverage per sequenced base pair compared to that obtained by direct sequencing of a leukocyte-filtered patient sample (Fig. 4A). The percentage of the 14 large chromosomes of *P. vivax* considered callable for samples that underwent SWGA fell within the range of that obtained by direct sequencing of leukocyte-filtered samples (Fig. 4B). Additionally, post-SWGA sequences yielded similar levels of mean base quality when normalized across 100-bp windows of various %GC contents in the reference genome compared to the results for leukocyte-filtered samples (Fig. 5).

10.1128/mBio.02257-16.5 FIG S5  Sequencing statistics for *Plasmodium vivax* clinical samples with various parasite densities. The percentage of reads that map to *P. vivax* Sal-1 (blue circles), the mean *P. vivax* genome coverage (green squares), and the percentage of the *P. vivax* genome covered by ≥5 reads (red triangles) are shown for all 18 samples amplified by SWGA, with sample parasitemia indicated on the *x* axis. Sequencing statistics were determined using the Genome Analysis Toolkit (GATK) DepthOfCoverage tool. Download FIG S5, TIF file, 31.4 MB. Copyright © 2017 Cowell et al. 2017 Cowell et al. This content is distributed under the terms of the Creative Commons Attribution 4.0 International license.

### Variant analysis.

To examine the utility of post-SWGA sequences for variant analysis, we called 45,821 single-nucleotide polymorphisms (SNPs) from the whole-blood and dried blood spot samples that were subjected to SWGA. For the whole-blood samples, an average of 14,463 SNPs was identified per sample, which is consistent with prior studies of *P. vivax* field isolates (10). Compared to the results for leukocyte-filtered samples, SNP characteristics such as SNP rate, transition-to-transversion (Ti/Tv) ratio, and nonsynonymous-to-synonymous ratio were nearly identical in the samples that underwent SWGA (Table 2).

**TABLE 2:** Comparison of single-nucleotide polymorphism characteristics of *Plasmodium vivax* sequences after selective whole-genome amplification (SWGA) and leukocyte filtration^a^

Sample preparation method	SNP characteristic					Nonsynonymous-to-synonymous ratio (SNP effect)
	Transition/transversion ratio	No. (%):			SNPs per base	
		Exonic	Intronic	Intergenic		
SWGA	1.41	20,865 (40)	3,714 (7)	25,178 (49)	0.002	1.63
Leukocyte filtration	1.36	18,365 (40)	3,029 (7)	23,157 (50)	0.002	1.74

a Samples for SWGA were obtained from Iquitos, Peru, and samples for leukocyte filtration were from a previously published study done in Madre de Dios, Peru (10).

In addition, the proportions of SNPs that were exonic, intronic, intergenic, or at 5′ and 3′ untranslated regions were similar between samples prepared using the two methods of *P. vivax* enrichment. We also detected SNPs in several known drug resistance genes previously detected in samples from Peru (10) and Colombia (8) in the whole-blood and dried blood spot samples (Table 3; see also Table S1 in the supplemental material), further validating the utility of sequences derived from SWGA for variant calling. This includes several intronic mutations around a putative chloroquine resistance transporter gene (*pvcrt*), in addition to coding mutations in the dihydrofolate reductase (*pvdhfr*), multidrug resistance protein 1 (*pvmdr1*), multidrug resistance protein 2 (*pvmrp2*), and dihydropteroate synthetase (*dhps*) genes.

10.1128/mBio.02257-16.9 TABLE S1  Drug resistance mutations in *Plasmodium vivax* sequences from dried blood spot DNA subjected to SWGA. Download TABLE S1, DOCX file, 0.01 MB. Copyright © 2017 Cowell et al. 2017 Cowell et al. This content is distributed under the terms of the Creative Commons Attribution 4.0 International license.

**TABLE 3:** Nonsynonymous SNPs in known drug resistance genes detected in *Plasmodium vivax* samples that underwent selective whole-genome amplification (SWGA)

Locus	Chromosome	Position	Reference allele^a^	Alternate allele^b^	Amino acid change^c^	No. of samples bearing indicated allele(no. confidently genotyped)
*pvcrt-0* (PVX_087980)	1	331151	T	C	Intron	17 (18)
		331819	G	A	Intron	11 (18)
		332453	T	C	Intron	18 (18)
		332874	A	C	Intron	18 (18)
*pvdhfr* (PVX_089950)	5	964763	C	G, A	Ser58Arg	12 (12)
		964939	G	A	Ser117Asn	16 (16)
*pvmdr1* (PVX_080100)	10	363223	A	G	Thr958Met	12 (12)
		363374	T	G	Met908Leu	11 (11)
		365435	C	A	Val221Leu	1 (12)
*pvmrp2* (PVX_124085)	14	2043859	G	C	Gln1407Glu	11 (13)
		2045050	C	T	Val1010Met	14 (15)
		2047090	G	A	Pro330Ser	1 (15)
		2047233	C	A	Arg282Met	13 (14)
		2047816	C	G	Glu88Gln	3 (18)
*dhps* (PVX_123230)	14	1257856	G	C	Ala383Gly	9 (15)
		1258389	C	T	Met205Ile	10 (12)

a Allele of the *P. vivax* Sal-1 reference genome at the indicated position.

b Allele found in *P. vivax* samples from Peru that underwent SWGA.

c Amino acid change resulting from base change from the reference to the alternate allele.

We also compared sample clonality estimates of post-SWGA sequences to microsatellite analyses on the same unamplified samples. We estimated the clonality of the 6 post-SWGA sequences with the highest coverage using the *F*_*ws*_ statistic, a measure of within-host diversity previously used to characterize multiplicity of infection in *Plasmodium falciparum* patient samples (Table 4) (5, 30). An *F*_*ws*_ score of ≥0.95 indicates low within-host diversity and infection with a single parasite, while an *F*_*ws*_ score of ≤0.70 is suggestive of a multiclonal infection. Microsatellite analysis on these same 6 unamplified samples indicated that all were clonal, except for sample 9, where the presence of 2 microsatellite markers at more than one position suggested that it could be a multiclonal sample. However, for all 6 post-SWGA sequences, the *F*_*ws*_ score was ≥0.95, suggesting that all were clonal infections. Thus, while SWGA does not introduce errors that lead to a falsely low *F*_*ws*_, it may lead to underestimations of clonality in multiclonal samples.

**TABLE 4:** Clonality estimates of samples that underwent selective whole-genome amplification compared to microsatellite analysis from the unamplified samples

Sample	*F_ws_*	No. of heterozygous SNPs	No. of microsatellite markers before SWGA in microsatellite locus:					
			*v23*	*v24*	*v25*	*v28*	*v30*	*v33*
1	0.98	377	266	170	102	85	149	153
2	0.97	446	266	170	102	85	149	163
5	0.97	372	266	171	102	86	150	153
6	0.98	326	266	170	102	86	149	153
8	0.98	300	282	167	102	94	136	134
9	0.97	354	282	163	99,94	86,118	143,150	151

**FIG 6:**
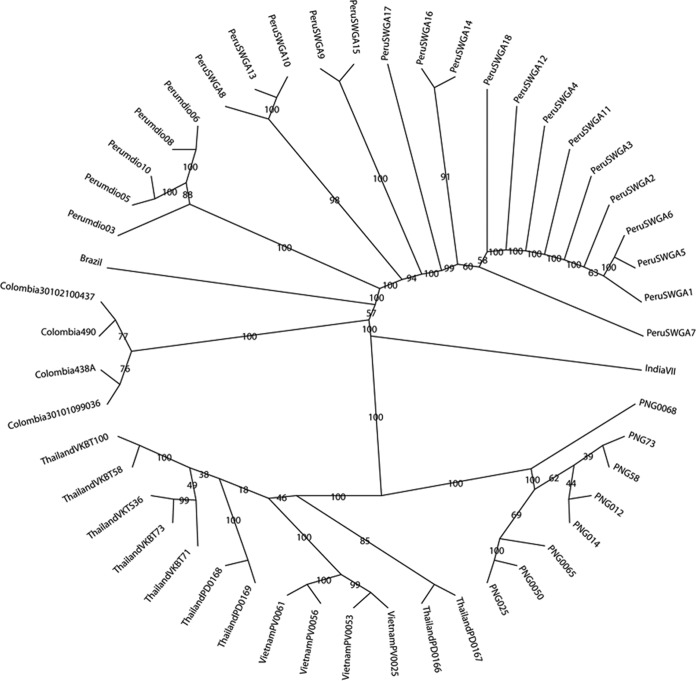
Neighbor-joining tree of *Plasmodium vivax* clinical samples from different regions of the world. The tree was constructed with core-genome-wide SNP data from *P. vivax* samples that underwent selective whole-genome amplification (Peru-SWGA-1 through Peru-SWGA-18) and *P. vivax* clinical samples from previously published studies that underwent leukocyte filtration (15) (Thailand-PD0169, Thailand-PD0168, Thailand-PD0166, Thailand-PD-0167, PNG-0050, PNG-0065, PNG-0068, Vietnam-PV0025, Vietnam-PV0053, Vietnam-PV0056, and Vietnam-PV0061), hybrid selection (17) (Colombia-30102100437, Colombia-490, Colombia-438−A, Colombia-30101099036, Thailand-VKTS-36, Thailand-VKBT-73, Thailand-VKBT-58, Thailand VKBT-71, Thailand-VKBT-100, PNG-73, PNG-58, PNG-012, PNG-014, and PNG-025), or adaption to growth in splenectomized monkeys (19) (BrazilI and IndiaVII) prior to sequencing. Bootstrap values are shown on each corresponding branch.

Finally, we constructed a neighbor-joining tree using core-genome SNPs to determine the relatedness of our samples to one another and to other *P. vivax* isolates from Peru and around the world (Fig. 6). In this tree, our samples, which were from the Iquitos region of Peru, clustered with one another and were most closely related to leukocyte-filtered samples from another region of Peru (10). They also exhibited the expected degree of relatedness to previously published *P. vivax* sequences derived from other leukocyte-filtered (15), hybrid-selected (17), and monkey-adapted (19) clinical samples from diverse areas of the world, further validating the use of post-SWGA sequences from downstream analyses.

## DISCUSSION

In this study, we validate SWGA as a cost-effective, robust method to enrich *P. vivax* gDNA from unprocessed whole-blood and dried blood spot clinical samples to improve the efficiency and decrease the cost of subsequent WGS. This is a method that can be applied to clinical samples infected with other malaria species, such as *P. malariae*, *P. ovale curtisi*, and *P. ovale wallikeri*, where parasite densities are low, and where there is no routine *ex vivo* culture (31), though species-specific primer sets would likely be required. SWGA utilizes readily available reagents, does not require processing at the time of sample collection, and can be performed in a simple, overnight reaction. While several methods have been used successfully to enrich *P. vivax* gDNA for WGS from infected whole-blood samples, most are resource and labor intensive. Short-term *ex vivo* culture of *P. vivax* isolates or adaptation to growth in monkeys produces a large amount of *P. vivax* DNA, but these methods require significant resources. Single-cell sequencing allows for highly sensitive dissection of multiclonal samples; however, this approach requires cryopreserved samples and specialized laboratory equipment (20). While leukocyte filtration is cost-effective and efficient, it is not always possible to perform at field sites with limited infrastructure, because samples require refrigeration within 6 hours to minimize white blood cell lysis and reduce irreversible contamination from human DNA. Hybrid selection is less labor intensive, but the production of the RNA baits used for capture requires either large amounts of *P. vivax* Sal-1 DNA or costly commercially synthesized RNA bait.

Using SWGA, we achieved a higher-than-average callable *P. vivax* genome than was obtained for leukocyte-depleted clinical samples sequenced at a similar depth. SWGA generally yielded the highest genome coverage for clinical samples with the highest parasite densities, consistent with our experience with *P. falciparum* (22). For the 12 samples with parasite densities of >5,000 parasites/µl (0.1% parasitemia), we were able to call, on average, 71.5% of the core genome, compared to 37% for the 6 samples with parasite densities of <5,000 parasites/µl. Increased sequencing effort is needed to obtain maximal genome coverage for samples with lower parasite densities (see Table S2 and Fig. S6 in the supplemental material). In these cases, the low genome coverage is likely the result of stochastic amplification of a small number of starting *P. vivax* genomes, which leads to very deep coverage of some genomic regions and little or no coverage of others (22). If maximal genome coverage is desired, sequential SWGA reactions with pvset1920 followed by pvset1 increase coverage slightly (3 to 5% 1× genome coverage) (see Fig. S7). Additionally, performing multiple independent SWGA reactions on a sample and combining the sequencing reads can improve genome coverage (4 to 12% 1× genome coverage) (see Fig. S8). Since multiple rounds of SWGA or pooling the products from multiple reactions increases the workload and expenses for a small improvement in genome coverage, we opted for a single amplification reaction with pvset1920 for our samples. However, these protocol modifications may be useful if high genome coverage is needed from samples with low parasitemia.

10.1128/mBio.02257-16.6 FIG S7  Combinatorial testing of SWGA primer sets on three *P. vivax*-infected patient samples. Each panel represents the results obtained for one of the three individual patient samples (samples 6, 2, and 4) after one round of SWGA with primer set pvset1920 (red) or pvset1 (blue), after two rounds of SWGA with the same primer set (dark red for pvset1920 and dark blue for pvset1), or after two rounds of SWGA with different primer sets (yellow for pvset1920 followed by pvset1 and light blue for pvset1 followed by pvset1920). For all primer set combinations, the percentage of the *P. vivax* reference genome covered at ≥1× is shown relative to the total sequencing depth (in millions of base pairs sequenced) generated on a MiSeq (Illumina). Download FIG S6, TIF file, 31.4 MB. Copyright © 2017 Cowell et al. 2017 Cowell et al. This content is distributed under the terms of the Creative Commons Attribution 4.0 International license.

10.1128/mBio.02257-16.7 FIG S8  Impact of multiple independent SWGA reactions per sample on *P. vivax* genome coverage. Each panel represents the results obtained for a single patient sample (samples 16, 7, and 14). The percentage of the *P. vivax* reference genome covered at ≥1× is shown relative to the total sequencing depth generated on an Illumina MiSeq (in millions of base pairs sequenced) for each individual SWGA reaction (red) or pooled reads from the two independent SWGA reactions (black). Download FIG S7, TIF file, 31.4 MB. Copyright © 2017 Cowell et al. 2017 Cowell et al. This content is distributed under the terms of the Creative Commons Attribution 4.0 International license.

10.1128/mBio.02257-16.8 FIG S6  *P. vivax* genome coverage for samples with high, medium, and low parasite densities. The percentages of the *P. vivax* reference genome covered at ≥1× are shown relative to the total sequencing depth generated on an Illumina HiSeq (in millions of base pairs sequenced) for samples with high (red), medium (green), and low (blue) parasite densities. Download FIG S8, TIF file, 11.3 MB. Copyright © 2017 Cowell et al. 2017 Cowell et al. This content is distributed under the terms of the Creative Commons Attribution 4.0 International license.

10.1128/mBio.02257-16.10 TABLE S2  Analysis of coverage relative to base pairs of sequencing effort for samples with high, medium, or low parasite densities. Download TABLE S2, DOCX file, 0.01 MB. Copyright © 2017 Cowell et al. 2017 Cowell et al. This content is distributed under the terms of the Creative Commons Attribution 4.0 International license.

One potential limitation of SWGA of *P. vivax* from clinical samples is the ability to amplify all clones in a multiclonal sample. One of our samples appeared to be multiclonal by microsatellite analysis of the unamplified gDNA, and yet, *F*_*ws*_ analysis of the post-SWGA sequence suggested that the sample was comprised of a single clone. It is possible that in this case, a majority clone was amplified preferentially over minority clones. Another possibility is that the microsatellite marker assessment may overestimate clonality. Analyses of additional multiclonal samples will be necessary to address this question. Another important limitation of SWGA is that copy number variant (CNV) detection is not possible on post-SWGA sequences. The uneven distribution of primer-targeted motifs in the target genome results in peaks and troughs in mean genome coverage that can confound CNV detection methods. Finally, SWGA requires long strands of gDNA for efficient amplification of the target genome and is unlikely to work well on degraded or ancient DNA samples.

Whole-genome analysis has the potential to reveal much about the biology and epidemiology of *P. vivax* infections. For example, comparison of recurrent infections using WGS can help distinguish relapse due to reactivation of hypnozoites from reinfection or drug resistance, an epidemiological distinction of public health importance. SWGA enables high-quality and cost-effective WGS of *P. vivax* from unprocessed blood samples that would otherwise be impossible or prohibitively expensive to sequence. Advanced technologies like SWGA will greatly facilitate future *P. vivax* whole-genome sequencing projects, thereby improving our ability to understand and combat the most widespread form of malaria.

## MATERIALS AND METHODS

### Patient sample collection and preparation.

The *P. vivax*-infected DNA sample used for initial testing of selective amplification primer sets (MRL2) was provided by the Malaria Reference Laboratory of the London School of Hygiene and Tropical Medicine, London, United Kingdom. The six dried blood spot samples used in this study were collected from patients with symptomatic *P. vivax* infection in Peru. Parasitemia was quantified with real-time PCR, and gDNA was extracted using a QIAamp DNA blood minikit (Qiagen).

Eighteen whole-blood samples used for additional testing and further sequencing analysis were derived from whole-blood samples collected from patients with symptomatic *P. vivax* infections from two sites around Iquitos, Peru, during a study conducted by U.S. Naval Medical Research Unit No. 6 (32). Thick blood smears were examined to identify the parasite species and to determine the level of parasitemia. Parasite density was calculated by counting the number of asexual parasites per 200 white blood cells in the thick smear (Assuming an average of 6,000 white blood cells per µl). Each blood smear was examined by two microscopists independently and by a third microscopist in the event of a discrepancy. The final parasite density was calculated as the average of the density readings from the two concordant microscopists. Whole-blood samples were collected in the field using EDTA-containing Vacutainer tubes. Samples were frozen and transported to the central laboratory until further processing. DNA was isolated from thawed whole blood using a QIAamp DNA blood minikit (Qiagen) following the manufacturer’s recommendations and as described elsewhere (33). Samples were subsequently resuspended in Tris-EDTA (TE) buffer, and gDNA was quantified using a Qubit 2.0 fluorometer.

### Primer design.

The initial set of pvset1 primers was designed as described previously (22). Primer set pvset1920 was designed using the *swga* program, which scores primer sets based on their selectivity and evenness of binding (measured using the Gini index) and, thus, automates and improves primer selection. The source code of *swga*, along with download links and documentation, are available at https://www.github.com/eclarke/swga. pvset1920 was designed to specifically amplify longer regions (>195,000 bp) of the *P. vivax* reference genome (Sal-1) that were GC rich (48.5 to 50.6%) and yielded low genome coverage following SWGA with pvset1. A total of 1,939 primer sets were identified that exhibited a minimum background binding distance of 25,000 bp and a maximum foreground binding distance of 37,000 bp. These were scored using *swga*’s composite primer scoring algorithm (Gini score × foreground mean/background mean), and the set with the lowest score (pvset1920) was chosen for testing.

pvset1 consists of the following 10 primers, with asterisks indicating phosphorothioate bonds that are necessary to prevent degradation by phi29: 5′-CGTTG*C*G-3′, 5′-TTTTTTC*G*C-3′, 5′-TCGTG*C*G-3′, 5′-CGTTTTTT*T*T-3′, 5′-TTTTTTTC*G*T-3′, 5′-CCGTT*C*G-3′, 5′-CGTTTC*G*T-3′, 5′-CGTTTC*G*C-3′, 5′-CGTTTT*C*G-3′, and 5′-TCGTTC*G*T-3′. pvset1920 consists of the following 12 primers: 5′-AACGAAGC*G*A-3′, 5′-ACGAAGCG*A*A-3′, 5′-ACGACGA*A*G-3′, 5′-ACGCGCA*A*C-3′, 5′-CAACGCG*G*T-3′, 5′-GACGAAA*C*G-3′, 5′-GCGAAAAA*G*G-3′, 5′-GCGAAGC*G*A-3′, 5′-GCGGAAC*G*A-3′, 5′-GCGTCGA*A*G-3′, 5′-GGTTAGCG*G*C-3′, and 5′-AACGAAT*C*G-3′.

### Selective whole-genome amplification.

Thirty to 70 ng of input DNA was added to a 50-µl reaction mixture containing 3.5 µM SWGA primers, 30 U phi29 DNA polymerase enzyme (New England Biolabs), 1× phi29 buffer (New England Biolabs), 4 mM dNTPs (Roche), 1% bovine serum albumin, and water. The reaction was carried out on a thermocycler with cycling conditions consisting of a ramp down from 35°C to 30°C (10 min per degree), 16 h at 30°C, 10 min at 65°C, and hold at 4°C. The samples were diluted 1:1 with DNase-free, RNase-free water and purified with AMPure XP beads (Beckman-Coulter) at a 1:1 ratio according to the manufacturer’s protocol. When a second round of selective amplification was performed, the reaction mixture contained 100 to 200 ng of the AMPure XP-purified product from the first reaction.

### Methylation digest.

One hundred twenty-five to 500 ng of gDNA extracted from *P. vivax*-infected whole-blood samples was digested with 5 units of FspEI (New England Biolabs) and 5 units of MspJI (New England Biolabs) enzymes in a 30-µl reaction mixture. A mock digest with an identical amount of gDNA and no enzymes was run in parallel. Samples were digested for 2 h at 37°C and then heat inactivated at 80°C for 15 min. For coverage analysis, rarefaction analysis was performed on Illumina MiSeq sequencing reads derived from samples digested with MspJI or FspEI (digest and SWGA) or mock digested with no enzyme prior to SWGA with primer set pvset1920. The coverage obtained by mapping 200,000 MiSeq sequencing reads (~25 million bp of sequencing depth) was compared between digested and mock-digested samples. *P* values were obtained using the two-tailed *t* test.

### Whole-genome sequencing.

The SWGA products and unamplified DNA used for primer set testing were sequenced on an Illumina MiSeq using a modified Nextera library preparation method. For HiSeq runs, next-generation-sequencing libraries of SWGA products were prepared using the Nextera XT DNA preparation kit (Illumina) according to the manufacturer’s protocol. These samples were pooled and clustered on a HiSeq 2500 (Illumina) in Rapid Run mode with 100-bp paired-end reads. For the coverage analysis of leukocyte-filtered samples, fastq files from a prior study of *P. vivax* field samples (10) were used.

Raw fastq files were aligned to the Sal-1 reference genome (PlasmoDB version 13, http://plasmodb.org/common/downloads/release-13.0/PvivaxSal1/fasta/data/) using the Burroughs-Wheeler Aligner (version 0.7.8) (34) and SAMtools (version 0.1.19) (35, 36) in the Platypus pipeline, as previously described (37). Picard (version 2.0.1) was used to remove unmapped reads, and the Genome Analysis Toolkit (GATK) (38) was used to realign the sequences around the indels. Picard’s CollectGcBiasMetrics tool was used to generate the GC bias plots. GATK’s DepthOfCoverage tool was used to determine the percentages of the total and core genomes covered by ≥5 reads, mean coverage, and coverage over the core genome. The coordinates of the *P. vivax* core genome, which excludes subtelomeric and hypervariable regions with significantly higher read mapping errors, was obtained from a recent analysis of hundreds of *P. vivax* sequences from clinical isolates (15).

For rarefaction analyses, sequences were aligned with smalt (Wellcome Trust Sanger Institute) and mapped with custom scripts in R (https://www.r-project.org/). To visualize base composition across chromosomes, plots were created in Geneious (version 9.1) (39) using the Sal-1 *P. vivax* reference sequences and 25-bp windows. Plots of chromosome coverage were created with IGVTools (version 2.3.40) (40, 41).

### Variant calling and analysis.

We followed the GATK’s best practices to call variants (42, 43). The aligned sequences were run through GATK’s HaplotypeCaller in “reference confidence” mode to create genomic variant call format (GVCF) files for each sample. This reference confidence model highlights areas of the genome that are likely to have variation and produces a comprehensive record of genotype likelihoods and annotations for each site. The samples were joint genotyped using the GenotypeGVCFs tool.

Variants were further filtered based on quality scores and sequencing bias statistics based on default parameters from GATK. SNPs were filtered out if they met any of the following criteria: quality depth (QD) of <2.0, mapping quality (MQ) of <50.0, Phred-scaled *P* value using Fisher’s exact test to detect strand bias (FS) of >60.0, symmetric odds ratio (SOR) of >4.0, *Z* score from Wilcoxon rank-sum test of alternative versus reference read-mapping qualities (MQRankSum) of <−12.5, and ReadPosRankSum (RPRS) of <−8.0. Variants were annotated using snpeff (version 4.2) (44).

*F*_*ws*_ of samples with the highest genome coverage was estimated using *moimix* (https://doi.org/10.5281/zenodo.58257), a package available through R. The package calculates the *F*_*ws*_ statistic using the equation *F*_*ws*_ = 1 − (*H_w_/H_s_*), where *H*_*w*_ is the within-host heterozygosity and *H*_*s*_ is the population-level heterozygosity (5, 30). The core *P. vivax* genome, as defined by Pearson et al. (15), was used for core-genome analysis. For microsatellite genotyping, five neutral microsatellite loci of significant variability in the Peruvian Amazon were typed in a previous study (32). If there was more than one marker at any given locus, the sample was considered multiclonal, per prior genotyping studies (45–47).

A neighbor-joining tree was constructed using SNPs from the core *P. vivax* genome from sequences obtained in this study, along with sequences from previously published studies that are available in the NCBI Short Read Archive. The Mdio samples were from a previous study conducted by our laboratory in Peru (10), and the rest of the sequences were obtained from two recent large-scale *P. vivax* sequencing studies (15, 17). In order to assess the phylogenetic relationships of sequenced isolates, we constructed a multiple sequence alignment from filtered SNPs called in GATK using an in-house Perl script. This alignment was used as the input for maximum-likelihood phylogenetic analysis in the randomized accelerated maximum-likelihood program (RAxML) (48) with 500 pseudoreplicates using the generalized time-reversible model, and the resulting tree was visualized in Dendroscope (49).

### Ethics approval and consent to participate.

The sample from Malaria Research Laboratories (MRL) was an anonymized DNA sample previously collected by the MRL and provided under the MRL’s remit to undertake epidemiological surveillance relevant to imported malaria in the United Kingdom. The protocol for the collection of field samples was approved by the Institutional Review Board of the U.S. Naval Medical Research Center (Protocol NMRCD.2005.0005) and the National Institutes of Health of Peru (protocol 009-2004) in compliance with all applicable federal regulations governing the protection of human subjects. All adult subjects provided written informed consent, and all children 8 to 17 years old provided verbal assent to participate in the study.

### Accession number(s).

The *P. vivax* genome Illumina sequencing reads of the 22 samples used for variant analysis in this study are available in the National Center for Biotechnology Information’s Sequence Read Archive with the study accession number SRP095853.

## References

[1] Singh B, Kim Sung L, Matusop A, Radhakrishnan A, Shamsul SS, Cox-Singh J, Thomas A, Conway DJ 2004 A large focus of naturally acquired Plasmodium knowlesi infections in human beings. Lancet 363:1017–1024. doi:10.1016/S0140-6736(04)15836-4. 15051281

[2] Calderaro A, Piccolo G, Gorrini C, Rossi S, Montecchini S, Dell’Anna ML, De Conto F, Medici MC, Chezzi C, Arcangeletti MC 2013 Accurate identification of the six human plasmodium spp. causing imported malaria, including Plasmodium ovale wallikeri and Plasmodium knowlesi. Malar J 12:321. doi:10.1186/1475-2875-12-321. 24034175 PMC3847200

[3] Sutherland CJ, Tanomsing N, Nolder D, Oguike M, Jennison C, Pukrittayakamee S, Dolecek C, Hien TT, do Rosário VE, Arez AP, Pinto J, Michon P, Escalante AA, Nosten F, Burke M, Lee R, Blaze M, Otto TD, Barnwell JW, Pain A, Williams J, White NJ, Day NP, Snounou G, Lockhart PJ, Chiodini PL, Imwong M, Polley SD 2010 Two nonrecombining sympatric forms of the human malaria parasite Plasmodium ovale occur globally. J Infect Dis 201:1544–1550. doi:10.1086/652240. 20380562

[4] 2015 World malaria report 2015. World Health Organization, Geneva, Switzerland.

[5] Manske M, Miotto O, Campino S, Auburn S, Almagro-Garcia J, Maslen G, O’Brien J, Djimde A, Doumbo O, Zongo I, Ouedraogo JB, Michon P, Mueller I, Siba P, Nzila A, Borrmann S, Kiara SM, Marsh K, Jiang H, Su XZ, Amaratunga C, Fairhurst R, Socheat D, Nosten F, Imwong M, White NJ, Sanders M, Anastasi E, Alcock D, Drury E, Oyola S, Quail MA, Turner DJ, Ruano-Rubio V, Jyothi D, Amenga-Etego L, Hubbart C, Jeffreys A, Rowlands K, Sutherland C, Roper C, Mangano V, Modiano D, Tan JC, Ferdig MT, Amambua-Ngwa A, Conway DJ, Takala-Harrison S, Plowe CV, Rayner JC 2012 Analysis of Plasmodium falciparum diversity in natural infections by deep sequencing. Nature 487:375–379. doi:10.1038/nature11174. 22722859 PMC3738909

[6] Borrmann S, Straimer J, Mwai L, Abdi A, Rippert A, Okombo J, Muriithi S, Sasi P, Kortok MM, Lowe B, Campino S, Assefa S, Auburn S, Manske M, Maslen G, Peshu N, Kwiatkowski DP, Marsh K, Nzila A, Clark TG 2013 Genome-wide screen identifies new candidate genes associated with artemisinin susceptibility in Plasmodium falciparum in Kenya. Sci Rep 3:3318. doi:10.1038/srep03318. 24270944 PMC3839035

[7] Miotto O, Almagro-Garcia J, Manske M, Macinnis B, Campino S, Rockett KA, Amaratunga C, Lim P, Suon S, Sreng S, Anderson JM, Duong S, Nguon C, Chuor CM, Saunders D, Se Y, Lon C, Fukuda MM, Amenga-Etego L, Hodgson AV, Asoala V, Imwong M, Takala-Harrison S, Nosten F, Su XZ, Ringwald P, Ariey F, Dolecek C, Hien TT, Boni MF, Thai CQ, Amambua-Ngwa A, Conway DJ, Djimdé AA, Doumbo OK, Zongo I, Ouedraogo JB, Alcock D, Drury E, Auburn S, Koch O, Sanders M, Hubbart C, Maslen G, Ruano-Rubio V, Jyothi D, Miles A, O’Brien J, Gamble C, Oyola SO 2013 Multiple populations of artemisinin-resistant Plasmodium falciparum in Cambodia. Nat Genet 45:648–655. doi:10.1038/ng.2624. 23624527 PMC3807790

[8] Winter DJ, Pacheco MA, Vallejo AF, Schwartz RS, Arevalo-Herrera M, Herrera S, Cartwright RA, Escalante AA 2015 Whole genome sequencing of field isolates reveals extensive genetic diversity in Plasmodium vivax from Colombia. PLoS Negl Trop Dis 9:e0004252. doi:10.1371/journal.pntd.0004252. 26709695 PMC4692395

[9] Carlton JM, Adams JH, Silva JC, Bidwell SL, Lorenzi H, Caler E, Crabtree J, Angiuoli SV, Merino EF, Amedeo P, Cheng Q, Coulson RM, Crabb BS, Del Portillo HA, Essien K, Feldblyum TV, Fernandez-Becerra C, Gilson PR, Gueye AH, Guo X, Kang’a S, Kooij TW, Korsinczky M, Meyer EV, Nene V, Paulsen I, White O, Ralph SA, Ren Q, Sargeant TJ, Salzberg SL, Stoeckert CJ, Sullivan SA, Yamamoto MM, Hoffman SL, Wortman JR, Gardner MJ, Galinski MR, Barnwell JW, Fraser-Liggett CM 2008 Comparative genomics of the neglected human malaria parasite Plasmodium vivax. Nature 455:757–763. doi:10.1038/nature07327. 18843361 PMC2651158

[10] Flannery EL, Wang T, Akbari A, Corey VC, Gunawan F, Bright AT, Abraham M, Sanchez JF, Santolalla ML, Baldeviano GC, Edgel KA, Rosales LA, Lescano AG, Bafna V, Vinetz JM, Winzeler EA 2015 Next-generation sequencing of Plasmodium vivax patient samples shows evidence of direct evolution in drug-resistance genes. Infect Dis 1:367–379. doi:10.1021/acsinfecdis.5b00049. PMC4692371 26719854

[11] Chan ER, Menard D, David PH, Ratsimbasoa A, Kim S, Chim P, Do C, Witkowski B, Mercereau-Puijalon O, Zimmerman PA, Serre D 2012 Whole genome sequencing of field isolates provides robust characterization of genetic diversity in Plasmodium vivax. PLoS Negl Trop Dis 6:e1811. doi:10.1371/journal.pntd.0001811. 22970335 PMC3435244

[12] Hester J, Chan ER, Menard D, Mercereau-Puijalon O, Barnwell J, Zimmerman PA, Serre D 2013 De novo assembly of a field isolate genome reveals novel Plasmodium vivax erythrocyte invasion genes. PLoS Negl Trop Dis 7:e2569. doi:10.1371/journal.pntd.0002569. 24340114 PMC3854868

[13] Venkatesan M, Amaratunga C, Campino S, Auburn S, Koch O, Lim P, Uk S, Socheat D, Kwiatkowski DP, Fairhurst RM, Plowe CV 2012 Using CF11 cellulose columns to inexpensively and effectively remove human DNA from Plasmodium falciparum-infected whole blood samples. Malar J 11:41. doi:10.1186/1475-2875-11-41. 22321373 PMC3295709

[14] Auburn S, Campino S, Clark TG, Djimde AA, Zongo I, Pinches R, Manske M, Mangano V, Alcock D, Anastasi E, Maslen G, Macinnis B, Rockett K, Modiano D, Newbold CI, Doumbo OK, Ouédraogo JB, Kwiatkowski DP 2011 An effective method to purify Plasmodium falciparum DNA directly from clinical blood samples for whole genome high-throughput sequencing. PLoS One 6:e22213. doi:10.1371/journal.pone.0022213. 21789235 PMC3138765

[15] Pearson RD, Amato R, Auburn S, Miotto O, Almagro-Garcia J, Amaratunga C, Suon S, Mao S, Noviyanti R, Trimarsanto H, Marfurt J, Anstey NM, William T, Boni MF, Dolecek C, Tran HT, White NJ, Michon P, Siba P, Tavul L, Harrison G, Barry A, Mueller I, Ferreira MU, Karunaweera N, Randrianarivelojosia M, Gao Q, Hubbart C, Hart L, Jeffery B, Drury E, Mead D, Kekre M, Campino S, Manske M, Cornelius VJ, MacInnis B, Rockett KA, Miles A, Rayner JC, Fairhurst RM, Nosten F, Price RN, Kwiatkowski DP 2016 Genomic analysis of local variation and recent evolution in Plasmodium vivax. Nat Genet 48:959–964. doi:10.1038/ng.3599. 27348299 PMC4966634

[16] Bright AT, Tewhey R, Abeles S, Chuquiyauri R, Llanos-Cuentas A, Ferreira MU, Schork NJ, Vinetz JM, Winzeler EA 2012 Whole genome sequencing analysis of Plasmodium vivax using whole genome capture. BMC Genomics 13:262. doi:10.1186/1471-2164-13-262. 22721170 PMC3410760

[17] Hupalo DN, Luo Z, Melnikov A, Sutton PL, Rogov P, Escalante A, Vallejo AF, Herrera S, Arévalo-Herrera M, Fan Q, Wang Y, Cui L, Lucas CM, Durand S, Sanchez JF, Baldeviano GC, Lescano AG, Laman M, Barnadas C, Barry A, Mueller I, Kazura JW, Eapen A, Kanagaraj D, Valecha N, Ferreira MU, Roobsoong W, Nguitragool W, Sattabonkot J, Gamboa D, Kosek M, Vinetz JM, González-Cerón L, Birren BW, Neafsey DE, Carlton JM 2016 Population genomics studies identify signatures of global dispersal and drug resistance in Plasmodium vivax. Nat Genet 48:953–958. doi:10.1038/ng.3588. 27348298 PMC5347536

[18] Auburn S, Marfurt J, Maslen G, Campino S, Ruano Rubio V, Manske M, Machunter B, Kenangalem E, Noviyanti R, Trianty L, Sebayang B, Wirjanata G, Sriprawat K, Alcock D, Macinnis B, Miotto O, Clark TG, Russell B, Anstey NM, Nosten F, Kwiatkowski DP, Price RN 2013 Effective preparation of Plasmodium vivax field isolates for high-throughput whole genome sequencing. PLoS One 8:e53160. doi:10.1371/journal.pone.0053160. 23308154 PMC3537768

[19] Neafsey DE, Galinsky K, Jiang RH, Young L, Sykes SM, Saif S, Gujja S, Goldberg JM, Young S, Zeng Q, Chapman SB, Dash AP, Anvikar AR, Sutton PL, Birren BW, Escalante AA, Barnwell JW, Carlton JM 2012 The malaria parasite Plasmodium vivax exhibits greater genetic diversity than Plasmodium falciparum. Nat Genet 44:1046–1050. doi:10.1038/ng.2373. 22863733 PMC3432710

[20] Nair S, Nkhoma SC, Serre D, Zimmerman PA, Gorena K, Daniel BJ, Nosten F, Anderson TJ, Cheeseman IH 2014 Single-cell genomics for dissection of complex malaria infections. Genome Res 24:1028–1038. doi:10.1101/gr.168286.113. 24812326 PMC4032849

[21] Larremore DB, Sundararaman SA, Liu W, Proto WR, Clauset A, Loy DE, Speede S, Plenderleith LJ, Sharp PM, Hahn BH, Rayner JC, Buckee CO 2015 Ape parasite origins of human malaria virulence genes. Nat Commun 6:8368. doi:10.1038/ncomms9368. 26456841 PMC4633637

[22] Sundararaman SA, Plenderleith LJ, Liu W, Loy DE, Learn GH, Li Y, Shaw KS, Ayouba A, Peeters M, Speede S, Shaw GM, Bushman FD, Brisson D, Rayner JC, Sharp PM, Hahn BH 2016 Genomes of cryptic chimpanzee plasmodium species reveal key evolutionary events leading to human malaria. Nat Commun 7:11078. doi:10.1038/ncomms11078. 27002652 PMC4804174

[23] Guggisberg AM, Sundararaman SA, Lanaspa M, Moraleda C, González R, Mayor A, Cisteró P, Hutchinson D, Kremsner PG, Hahn BH, Bassat Q, Odom AR 2016 Whole genome sequencing to evaluate the resistance landscape following antimalarial treatment failure with fosmidomycin-clindamycin. J Infect Dis 214:1085–1091. doi:10.1093/infdis/jiw304. 27443612 PMC5021231

[24] Leichty AR, Brisson D 2014 Selective whole genome amplification for resequencing target microbial species from complex natural samples. Genetics 198:473–481. doi:10.1534/genetics.114.165498. 25096321 PMC4196606

[25] McCutchan TF, Dame JB, Miller LH, Barnwell J 1984 Evolutionary relatedness of plasmodium species as determined by the structure of DNA. Science 225:808–811. doi:10.1126/science.6382604. 6382604

[26] Morin JA, Cao FJ, Lázaro JM, Arias-Gonzalez JR, Valpuesta JM, Carrascosa JL, Salas M, Ibarra B 2012 Active DNA unwinding dynamics during processive DNA replication. Proc Natl Acad Sci U S A 109:8115–8120. doi:10.1073/pnas.1204759109. 22573817 PMC3361432

[27] Schwartz A, Baidjoe A, Rosenthal PJ, Dorsey G, Bousema T, Greenhouse B 2015 The effect of storage and extraction methods on amplification of Plasmodium falciparum DNA from dried blood spots. Am J Trop Med Hyg 92:922–925. doi:10.4269/ajtmh.14-0602. 25758652 PMC4426578

[28] Cohen-Karni D, Xu D, Apone L, Fomenkov A, Sun Z, Davis PJ, Kinney SR, Yamada-Mabuchi M, Xu SY, Davis T, Pradhan S, Roberts RJ, Zheng Y 2011 The MspJI family of modification-dependent restriction endonucleases for epigenetic studies. Proc Natl Acad Sci U S A 108:11040–11045. doi:10.1073/pnas.1018448108. 21690366 PMC3131316

[29] Oyola SO, Gu Y, Manske M, Otto TD, O’Brien J, Alcock D, Macinnis B, Berriman M, Newbold CI, Kwiatkowski DP, Swerdlow HP, Quail MA 2013 Efficient depletion of host DNA contamination in malaria clinical sequencing. J Clin Microbiol 51:745–751. doi:10.1128/JCM.02507-12. 23224084 PMC3592063

[30] Auburn S, Campino S, Miotto O, Djimde AA, Zongo I, Manske M, Maslen G, Mangano V, Alcock D, MacInnis B, Rockett KA, Clark TG, Doumbo OK, Ouédraogo JB, Kwiatkowski DP 2012 Characterization of within-host Plasmodium falciparum diversity using next-generation sequence data. PLoS One 7:e32891. doi:10.1371/journal.pone.0032891. 22393456 PMC3290604

[31] Ansari HR, Templeton TJ, Subudhi AK, Ramaprasad A, Tang J, Lu F, Naeem R, Hashish Y, Oguike MC, Benavente ED, Clark TG, Sutherland CJ, Barnwell JW, Culleton R, Cao J, Pain A 2016 Genome-scale comparison of expanded gene families in Plasmodium ovale wallikeri and Plasmodium ovale curtisi with Plasmodium malariae and with other plasmodium species. Int J Parasitol 46:685–696. doi:10.1016/j.ijpara.2016.05.009. 27392654

[32] Durand S, Cabezas C, Lescano AG, Galvez M, Gutierrez S, Arrospide N, Alvarez C, Santolalla ML, Bacon DJ, Graf PC 2014 Efficacy of three different regimens of primaquine for the prevention of relapses of Plasmodium vivax malaria in the Amazon Basin of Peru. Am J Trop Med Hyg 91:18–26. doi:10.4269/ajtmh.13-0053. 24752682 PMC4080559

[33] Baldeviano GC, Okoth SA, Arrospide N, Gonzalez RV, Sánchez JF, Macedo S, Conde S, Tapia LL, Salas C, Gamboa D, Herrera Y, Edgel KA, Udhayakumar V, Lescano AG 2015 Molecular epidemiology of Plasmodium falciparum malaria outbreak, Tumbes, Peru, 2010-2012. Emerg Infect Dis 21:797–803. doi:10.3201/eid2105.141427. 25897626 PMC4412223

[34] Li H 2013 Aligning sequence reads, clone sequences and assembly contigs with BWA-MEM. Arxiv arXiv:1303.3997 [q-bio.GN] https://arxiv.org/abs/1303.3997.

[35] Li H, Handsaker B, Wysoker A, Fennell T, Ruan J, Homer N, Marth G, Abecasis G, Durbin R, 1000 Genome Project Data Processing Subgroup 2009 The sequence alignment/map format and SAMtools. Bioinformatics 25:2078–2079. doi:10.1093/bioinformatics/btp352. 19505943 PMC2723002

[36] Li H 2011 A statistical framework for SNP calling, mutation discovery, association mapping and population genetical parameter estimation from sequencing data. Bioinformatics 27:2987–2993. doi:10.1093/bioinformatics/btr509. 21903627 PMC3198575

[37] Manary MJ, Singhakul SS, Flannery EL, Bopp SE, Corey VC, Bright AT, McNamara CW, Walker JR, Winzeler EA 2014 Identification of pathogen genomic variants through an integrated pipeline. BMC Bioinformatics 15:63. doi:10.1186/1471-2105-15-63. 24589256 PMC3945619

[38] McKenna A, Hanna M, Banks E, Sivachenko A, Cibulskis K, Kernytsky A, Garimella K, Altshuler D, Gabriel S, Daly M, DePristo MA 2010 The genome analysis toolkit: a MapReduce framework for analyzing next-generation DNA sequencing data. Genome Res 20:1297–1303. doi:10.1101/gr.107524.110. 20644199 PMC2928508

[39] Kearse M, Moir R, Wilson A, Stones-Havas S, Cheung M, Sturrock S, Buxton S, Cooper A, Markowitz S, Duran C, Thierer T, Ashton B, Meintjes P, Drummond A 2012 Geneious Basic: an integrated and extendable desktop software platform for the organization and analysis of sequence data. Bioinformatics 28:1647–1649. doi:10.1093/bioinformatics/bts199. 22543367 PMC3371832

[40] Robinson JT, Thorvaldsdóttir H, Winckler W, Guttman M, Lander ES, Getz G, Mesirov JP 2011 Integrative genomics viewer. Nat Biotechnol 29:24–26. doi:10.1038/nbt.1754. 21221095 PMC3346182

[41] Thorvaldsdóttir H, Robinson JT, Mesirov JP 2013 Integrative genomics viewer (IGV): high-performance genomics data visualization and exploration. Brief Bioinform 14:178–192. doi:10.1093/bib/bbs017. 22517427 PMC3603213

[42] DePristo MA, Banks E, Poplin R, Garimella KV, Maguire JR, Hartl C, Philippakis AA, del Angel G, Rivas MA, Hanna M, McKenna A, Fennell TJ, Kernytsky AM, Sivachenko AY, Cibulskis K, Gabriel SB, Altshuler D, Daly MJ 2011 A framework for variation discovery and genotyping using next-generation DNA sequencing data. Nat Genet 43:491–498. doi:10.1038/ng.806. 21478889 PMC3083463

[43] Van der Auwera GA, Carneiro MO, Hartl C, Poplin R, Del Angel G, Levy-Moonshine A, Jordan T, Shakir K, Roazen D, Thibault J, Banks E, Garimella KV, Altshuler D, Gabriel S, DePristo MA 2013 From FastQ data to high confidence variant calls: the genome analysis toolkit best practices pipeline. Curr Protoc Bioinformatics 43:11.10.1–11.10.33. doi:10.1002/0471250953.bi1110s43. 25431634 PMC4243306

[44] Cingolani P, Platts A, Wang LL, Coon M, Nguyen T, Wang L, Land SJ, Lu X, Ruden DM 2012 A program for annotating and predicting the effects of single nucleotide polymorphisms, SnpEff: SNPs in the genome of Drosophila melanogaster strain w1118; iso-2; iso-3. Fly 6:80–92. doi:10.4161/fly.19695. 22728672 PMC3679285

[45] Karunaweera ND, Ferreira MU, Munasinghe A, Barnwell JW, Collins WE, King CL, Kawamoto F, Hartl DL, Wirth DF 2008 Extensive microsatellite diversity in the human malaria parasite Plasmodium vivax. Gene 410:105–112. doi:10.1016/j.gene.2007.11.022. 18226474

[46] Imwong M, Sudimack D, Pukrittayakamee S, Osorio L, Carlton JM, Day NP, White NJ, Anderson TJ 2006 Microsatellite variation, repeat array length, and population history of Plasmodium vivax. Mol Biol Evol 23:1016–1018. doi:10.1093/molbev/msj116. 16507919

[47] de Souza AM, de Araújo FC, Fontes CJ, Carvalho LH, de Brito CF, de Sousa TN 2015 Multiple-clone infections of Plasmodium vivax: definition of a panel of markers for molecular epidemiology. Malar J 14:330. doi:10.1186/s12936-015-0846-5. 26303668 PMC4548710

[48] Stamatakis A 2014 RAxML version 8: a tool for phylogenetic analysis and post-analysis of large phylogenies. Bioinformatics 30:1312–1313. doi:10.1093/bioinformatics/btu033. 24451623 PMC3998144

[49] Huson DH, Scornavacca C 2012 Dendroscope 3: an interactive tool for rooted phylogenetic trees and networks. Syst Biol 61:1061–1067. doi:10.1093/sysbio/sys062. 22780991

