# Supplementary figures and images for: Selective Whole-Genome Amplification Is a Robust Method That Enables Scalable Whole-Genome Sequencing of Plasmodium vivax from Unprocessed Clinical Samples
Source: mBio. 2017 Feb 7;8(1):e02257-16. doi: 10.1128/mBio.02257-16 (PMC5296604; doi:10.1128/mBio.02257-16)

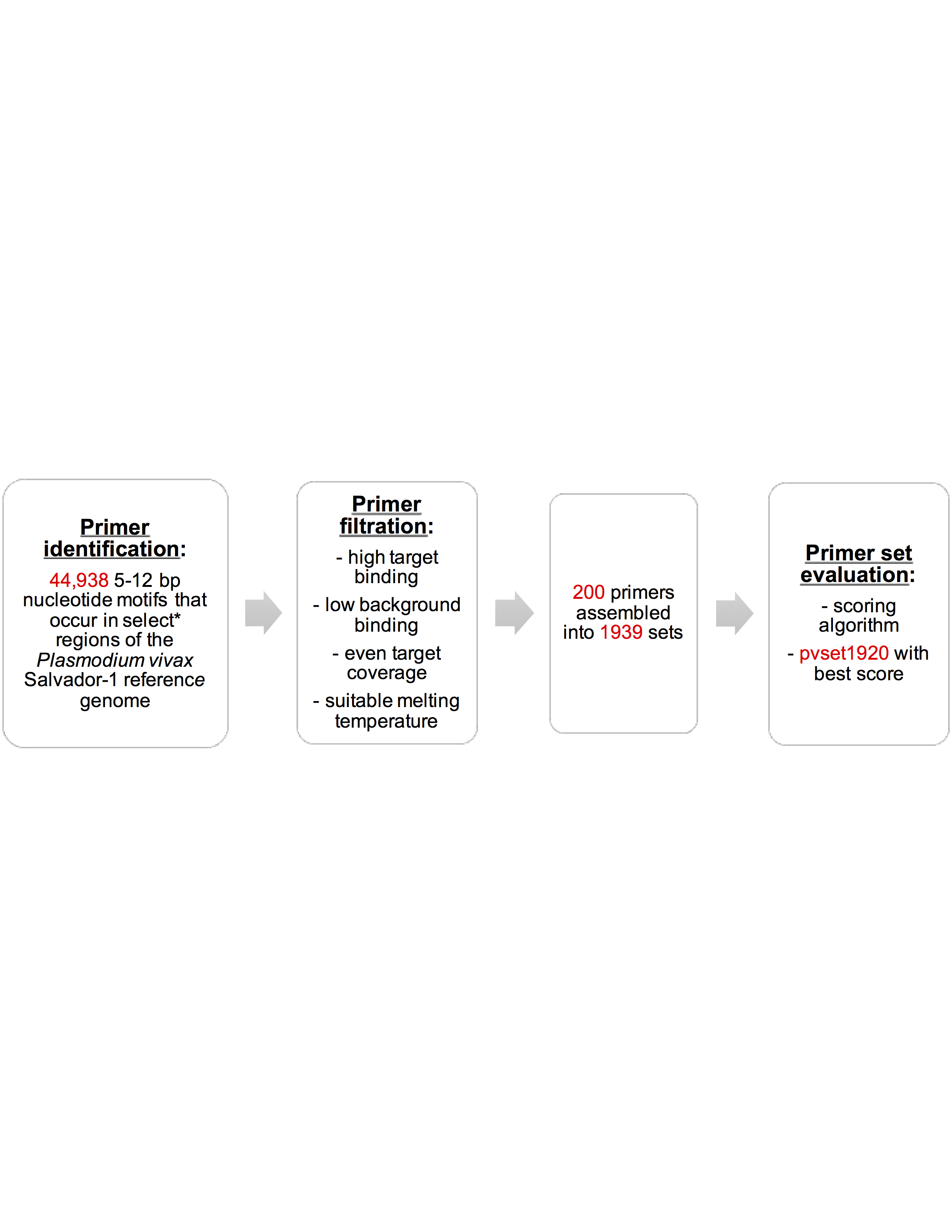

Supplement: FIG S2 [file mbo001173166sf2.tif]

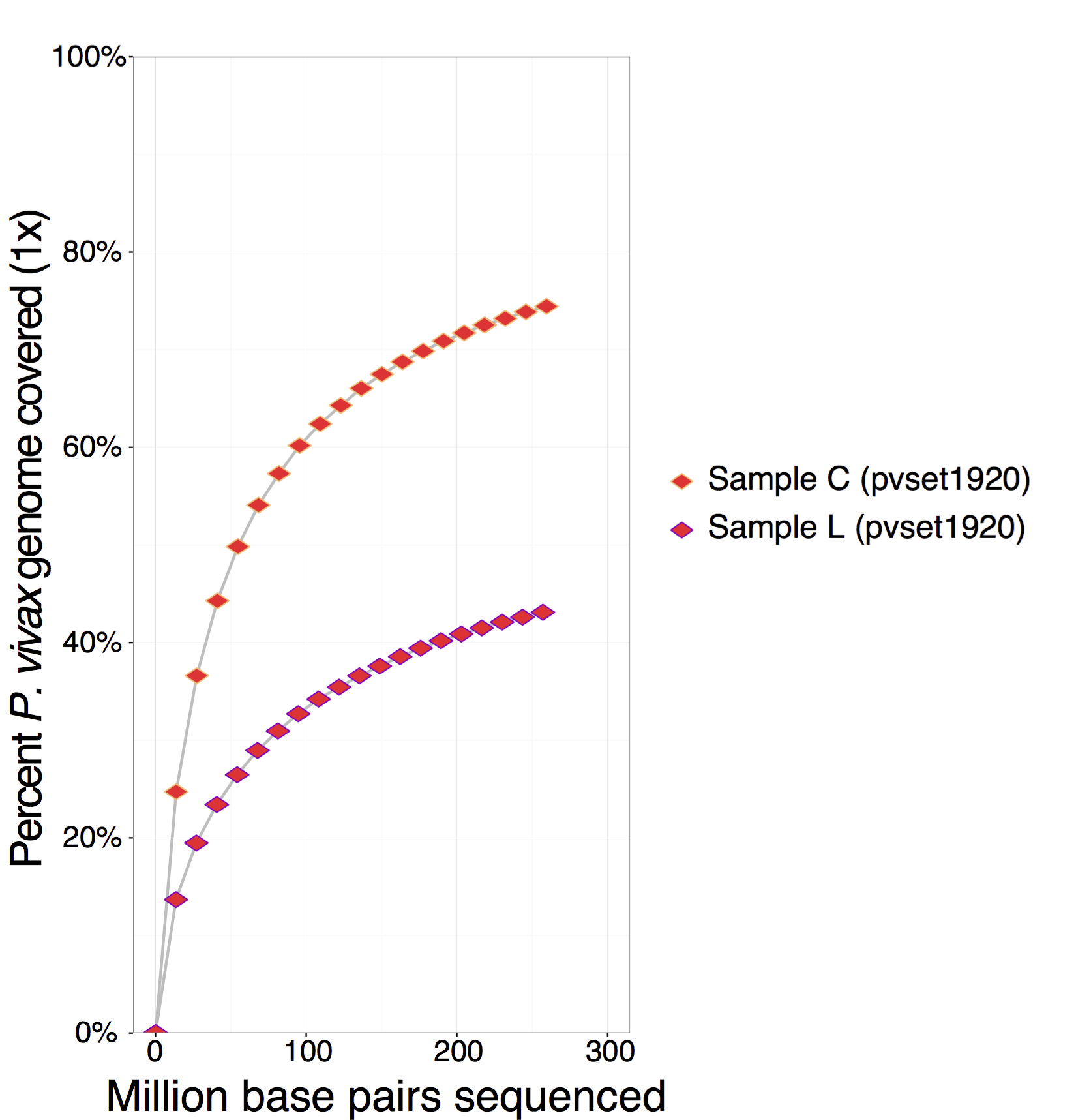

Supplement: FIG S3 [file mbo001173166sf3.tif]

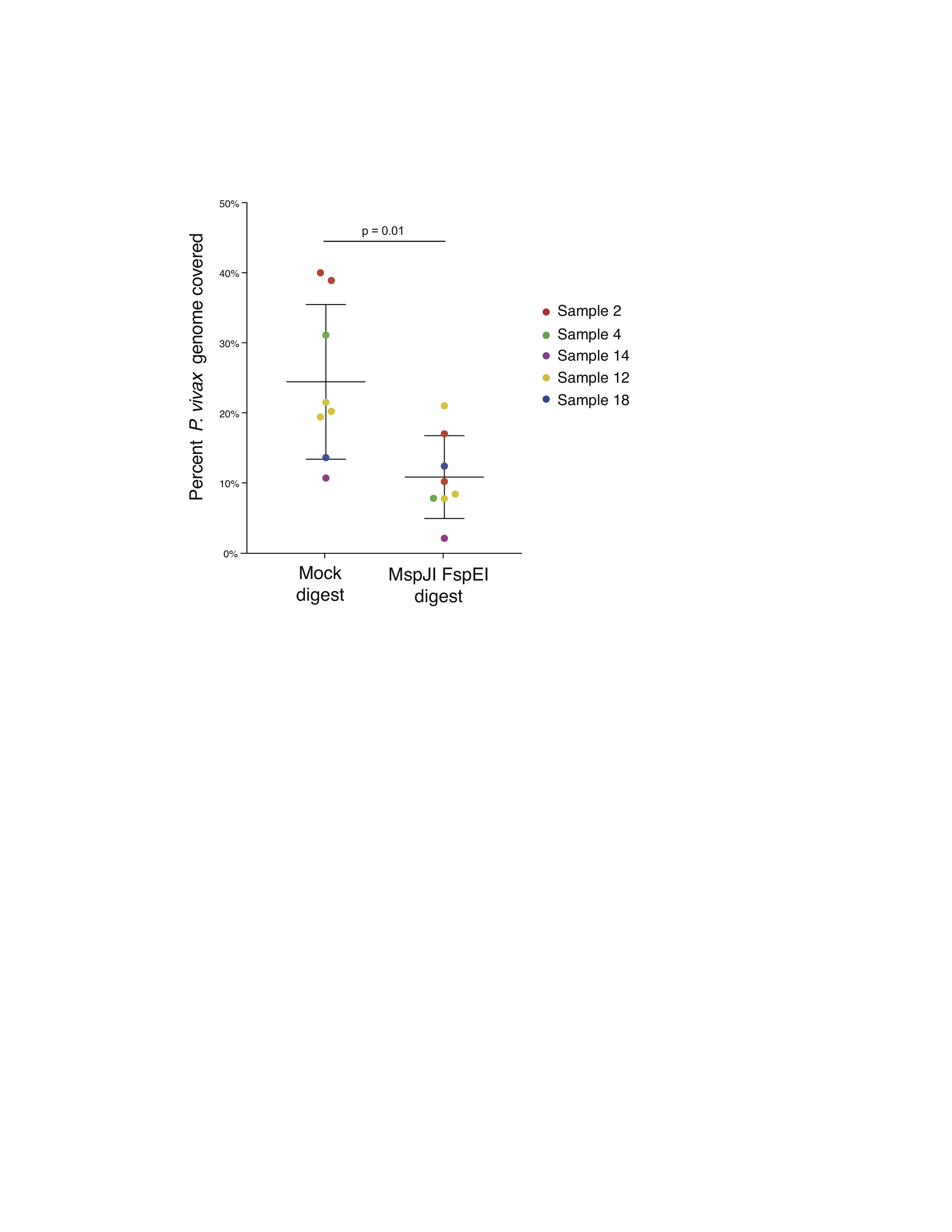

Supplement: FIG S4 [file mbo001173166sf4.tif]

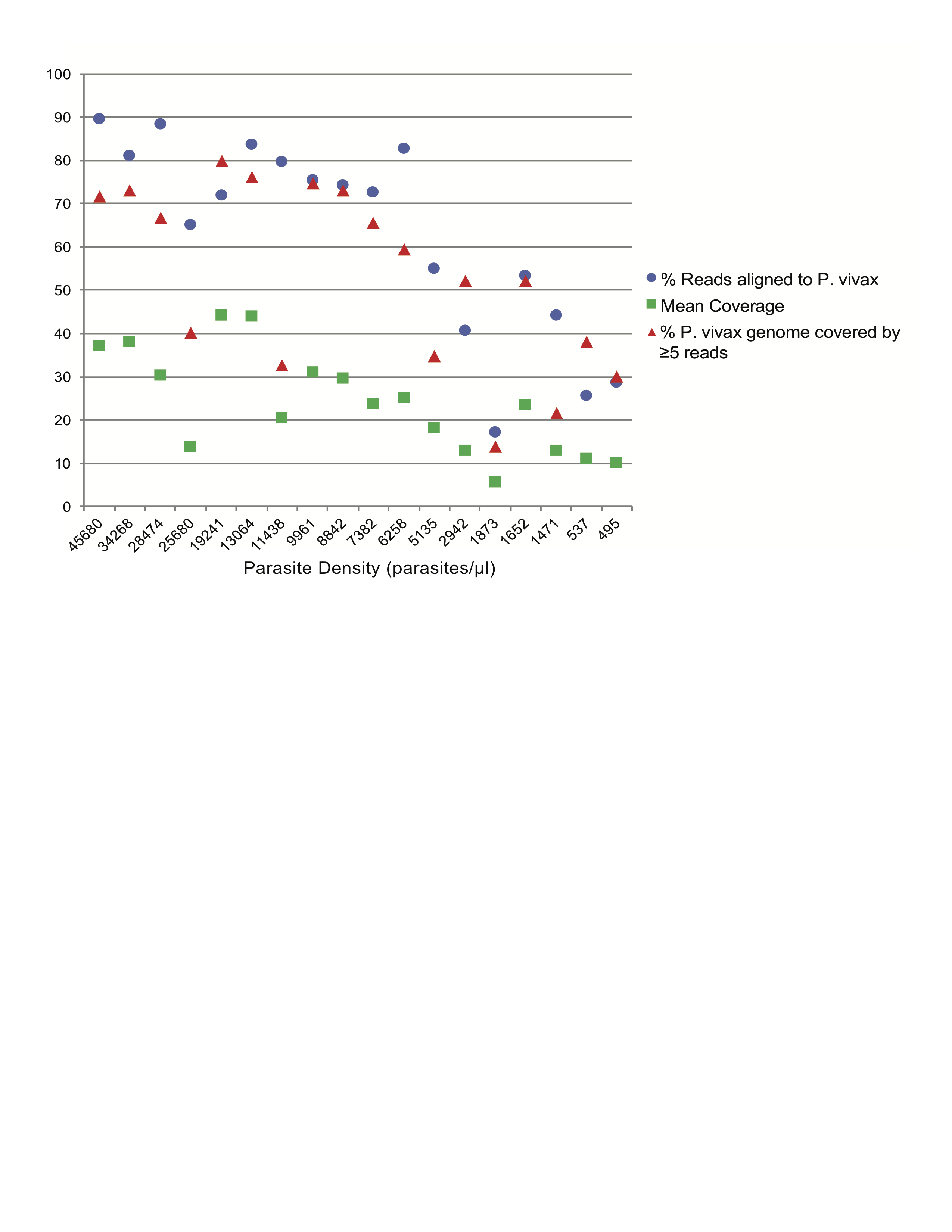

Supplement: FIG S5 [file mbo001173166sf5.tif]

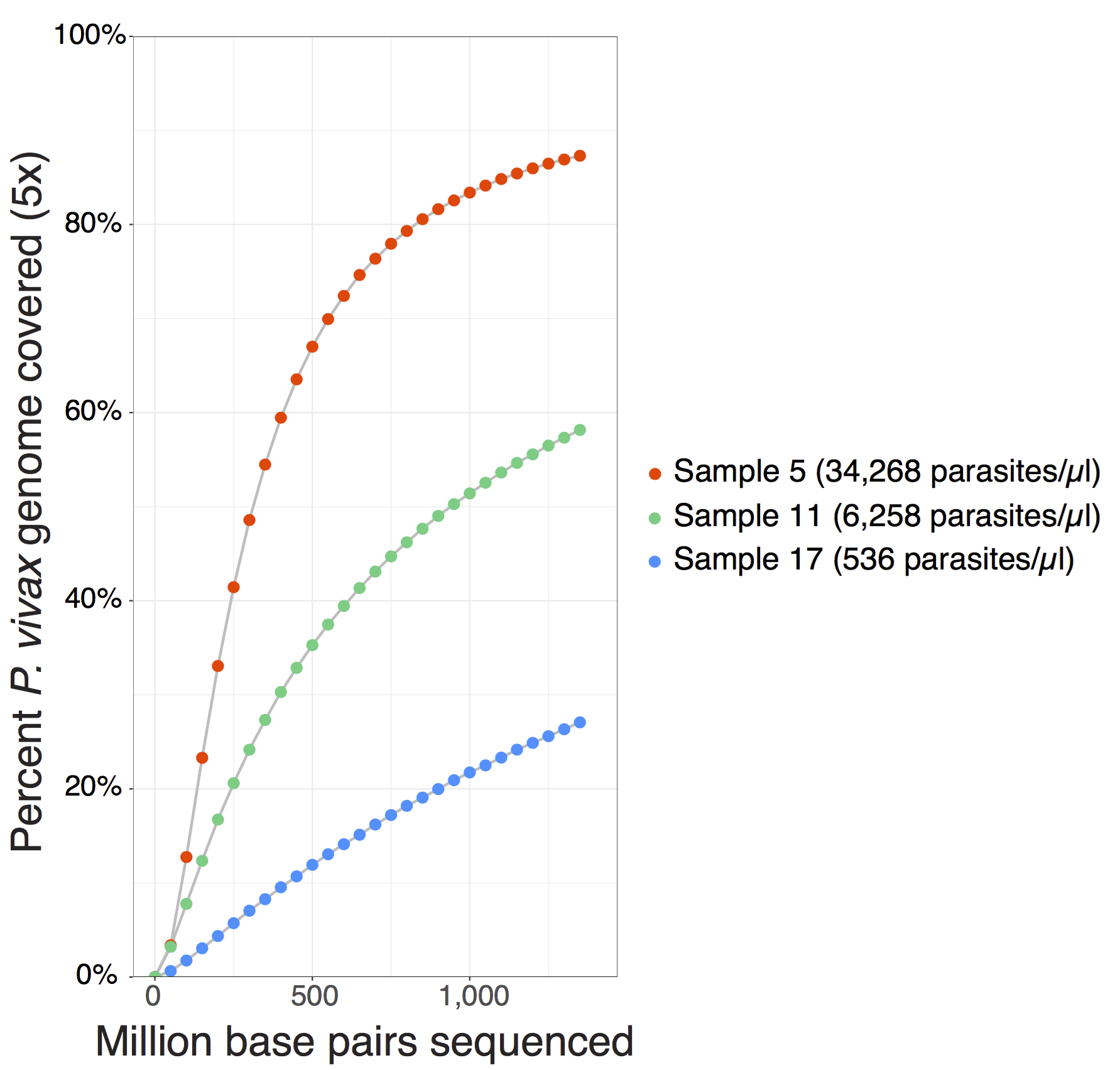

Supplement: FIG S7 [file mbo001173166sf6.tif]

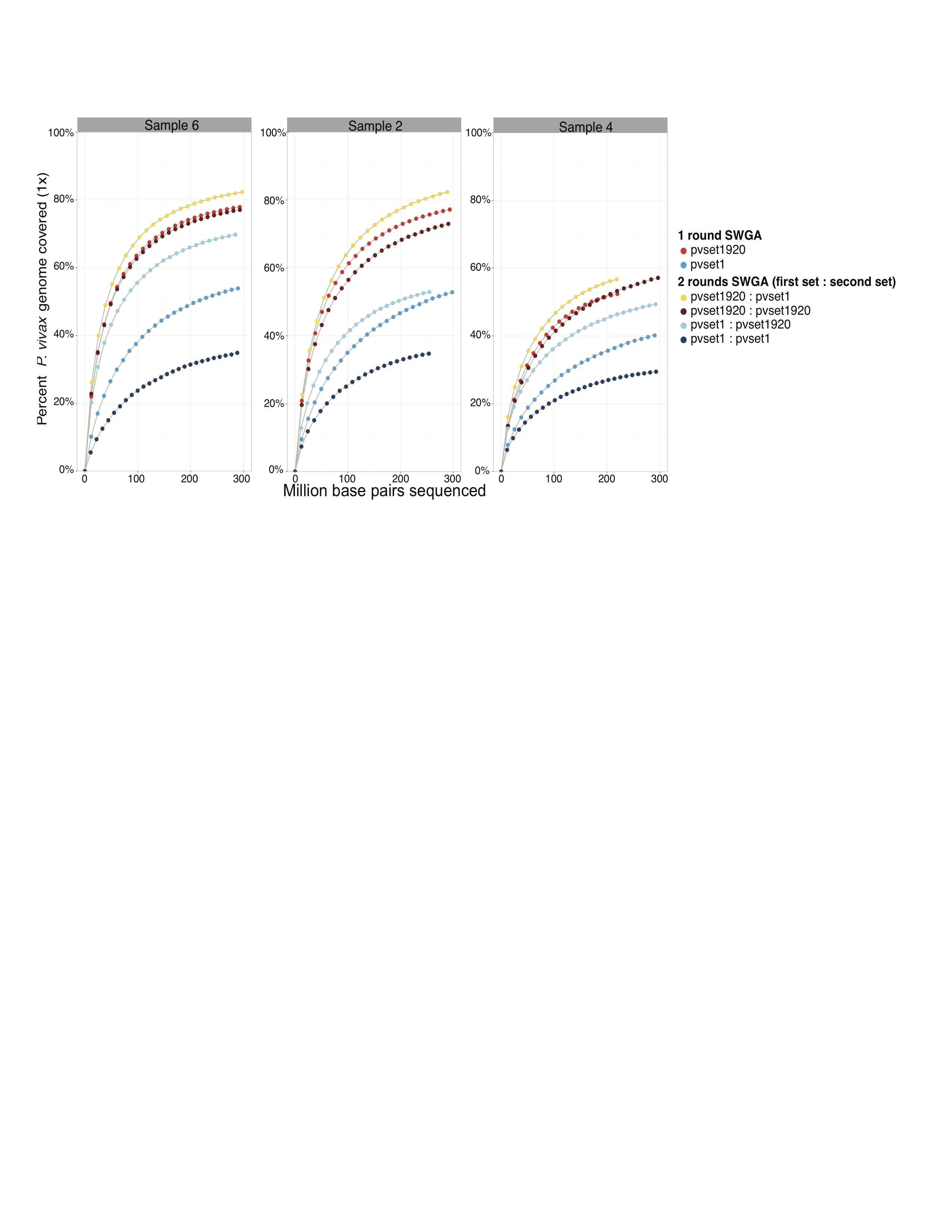

Supplement: FIG S8 [file mbo001173166sf7.tif]

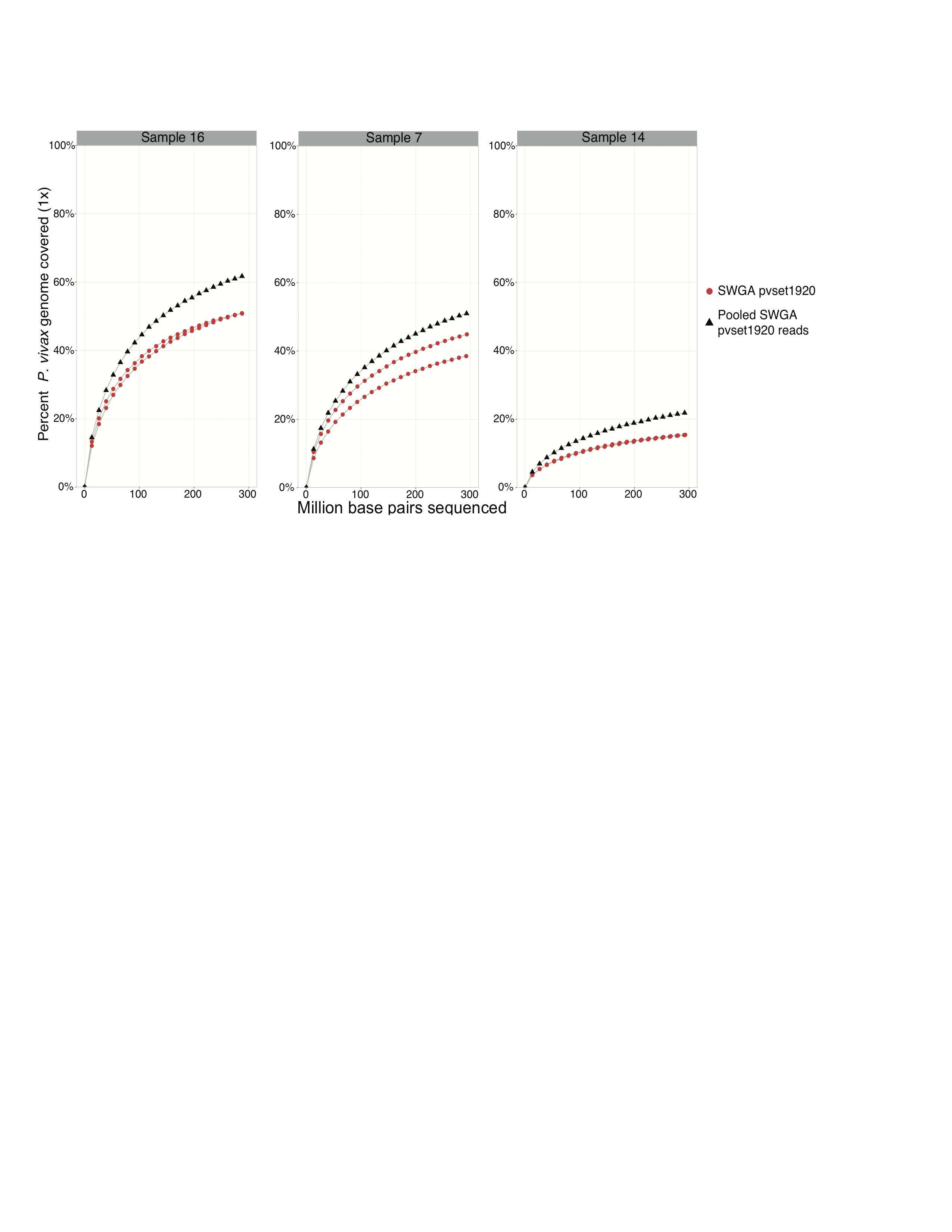

Supplement: FIG S6 [file mbo001173166sf8.tif]
