# Supplementary material for: Selective Whole-Genome Amplification Is a Robust Method That Enables Scalable Whole-Genome Sequencing of Plasmodium vivax from Unprocessed Clinical Samples
Source: mBio. 2017 Feb 7;8(1):e02257-16. doi: 10.1128/mBio.02257-16 (PMC5296604; doi:10.1128/mBio.02257-16)
Supplement: TABLE S1 [file mbo001173166st1.docx]

Supplementary Table 1. Drug resistance mutations in *Plasmodium vivax* sequences from dried blood spot DNA subjected to SWGA.

| **Locus** | **Chr** | **Position** | **Ref** | **Alt** | **Amino Acid** | **Samples*** |
| --- | --- | --- | --- | --- | --- | --- |
| *pvcrt-0* (PVX_087980) | 1 | 331151 | T | C | Intron | 3 (4) |
|  |  | 331819 | G | A | Intron | 1 (4) |
|  |  | 332453 | T | C | Intron | 4 (4) |
|  |  | 332874 | A | C | Intron | 4 (4) |
| *pvdhfr* (PVX_089950) | 5 | 964762 | G | A | Ser58Asn | 1 (4) |
|  |  | 964763 | C | G,A | Ser58Arg | 3 (3) |
|  |  | 964939 | G | A | Ser117Asn | 3 (3) |
| *pvmdr1* (PVX_080100) | 10 | 363223 | A | G | Thr958Met | 4 (4) |
|  |  | 363374 | T | G | Met908Leu | 2 (3) |
| *pvmrp2* (PVX_124085) | 14 | 2043859 | G | C | Gln1407Glu | 1 (3) |
|  |  | 2044327 | T | A | Asn1251Tyr | 1 (3) |
|  |  | 2045050 | C | T | Val1010Met | 4 (4) |
|  |  | 2047233 | C | A | Arg282Met | 2 (2) |
|  |  | 2047816 | C | G | Glu88Gln | 2 (4) |
|  |  | 2047893 | C | T | Cys62Tyr | 1 (4) |
| *dhps* (PVX_123230) | 14 | 1257856 | G | C | Ala383Gly | 1 (4) |
|  |  | 1258389 | C | T | Met205Ile | 2 (3) |

*Samples shows the number of samples confidently genotyped (in parentheses) and the number bearing the indicated allele.
