# Supplementary material for: Selective Whole-Genome Amplification Is a Robust Method That Enables Scalable Whole-Genome Sequencing of Plasmodium vivax from Unprocessed Clinical Samples
Source: mBio. 2017 Feb 7;8(1):e02257-16. doi: 10.1128/mBio.02257-16 (PMC5296604; doi:10.1128/mBio.02257-16)
Supplement: TABLE S2 [file mbo001173166st2.docx]

Supplementary Table 2. Analysis of coverage relative to base pairs of sequencing effort for samples with high, medium, or low parasites densities.

| **Parasite density range (parasites/µl)** | **Average % reads aligned to *P. vivax*** | **Average % core genome callable** | **Average base pairs sequenced (billions)** | **Base pairs sequencing recommended for maximal genome coverage (billions)** |
| --- | --- | --- | --- | --- |
| ≥10,000 | 79.8 | 62.9 | 1.6 | 1.5 |
| ≥5,000 - 10,000 | 72.0 | 61.5 | 1.4 | 1.5-2.5 |
| <5,000 | 34.8 | 34.6 | 1.5 | >5.0 |
